# Navigating value complexity in care pathway development: a qualitative case study

**DOI:** 10.1136/bmjopen-2024-098157

**Published:** 2025-08-13

**Authors:** Mildred Visser, Marleen de Mul, Kees Ahaus, Anne Marie Weggelaar-Jansen

**Affiliations:** 1 Erasmus School of Health Policy & Management, Department Health Services Management & Organization, Erasmus University Rotterdam, Rotterdam, The Netherlands; 2 Clinical Informatics, Eindhoven University of Technology, Eindhoven, The Netherlands; 3 Tranzo, Tilburg School of Social and Behavioural Sciences, Tilburg University, Tilburg, The Netherlands

**Keywords:** Health Services, Quality in health care, Delivery of Health Care, Integrated, Change management, QUALITATIVE RESEARCH, Quality Improvement

## Abstract

**Abstract:**

**Objectives:**

Care pathways (CPs) are widely used to standardise and improve healthcare delivery. However, CP development is often shaped by value (or normative) complexity. This study empirically explores how value complexity unfolds in a CP development programme.

**Design:**

A qualitative single-case study was conducted as part of a 2-year action research programme. The study followed a ‘research-follow-action’ strategy, in which action and learning occurred during the programme phase, followed by retrospective analysis using Greenhalgh *et al*’s ‘rules of thumb’ as a reflective lens.

**Setting:**

A Dutch specialised rehabilitation hospital (13 sites, 800 employees approximately, ~16 000 patients annually). In three CP development cycles, 11 multidisciplinary teams were guided in CP development in a quality collaborative approach.

**Participants:**

26 respondents participated in 44 reflective conversations; 19 respondents completed reflective questionnaires and 169 participatory observation reports were included. Participants were purposively sampled and included representatives from the leadership triad (rehabilitation physicians, managers and healthcare professionals) and members of senior management involved in CP development.

**Results:**

Two overarching themes were identified: goal (mis)alignment and prolonged decision-making processes negatively impacted motivation and impeded CP development. Goal alignment between stakeholders was hindered by shifting organisational priorities, creating tensions between improving care quality and ensuring financial viability. Decision-making was challenged by role uncertainty and the complexities of multidisciplinary collaboration in CP development teams. Reflective dialogues, small-scale experimentation and financial modelling supported teams in navigating these tensions to varying degrees.

**Conclusions:**

This study illustrates how value complexity unfolds in CP development and underscores the importance of ongoing stakeholder management, reflectivity, formative evaluation and dialogue. Greenhalgh *et al*’s rules of thumb provided interpretive value in exploring these complexities but require a solid theoretical understanding and an awareness of the rules’ interrelationships. A complexity-informed approach integrating ongoing reflection and adaptability can enrich CP development methodologies, enabling healthcare professionals and action researchers to engage constructively with value complexity in complex change processes. Further research is needed to develop and implement practical strategies for enhancing stakeholder engagement and decision-making in diverse healthcare settings.

**STRENGTHS AND LIMITATIONS OF THIS STUDY**

- In-depth embedded qualitative approach provided rich insights into value complexity in the context of care pathway development processes.
- The longitudinal nature of the study (2-year action research programme) allowed observation of evolving complexities.
- Triangulation of data sources (reflective conversations, questionnaires and participatory observations) enhanced credibility.
- The single-case study design may limit transferability to other healthcare settings.
- Potential for researcher bias due to the dual role of researcher/facilitator, mitigated by reflexive practices.

## Introduction

 Care pathways (CPs) are widely used to standardise and improve care quality and safety.^1^,^4^ They integrate evidence-based knowledge into routine practices, improving health outcomes and efficiency, fostering innovation in care delivery and ensuring continuity of care.^1 2 4^ The definition and purpose of CPs has been subject to debate in recent decades. In 2007, Vanhaecht^5^ (p137) defined a CP as ‘a complex intervention for the mutual decision-making and organisation of care processes for a well-defined group of patients during a well-defined period’. Vanhaecht *et al*^2^ later conceptualised CPs as a concept for patient-focused care, encompassing three dimensions: (1) the model (eg, a chain, hub or web model, depending on the level of care predictability and professional consensus), (2) the process (ie, methodology for developing and implementing well-organised care to improve quality and efficiency) and (3) the product (eg, a quality document or process flow diagram). Against this background, this study explores how CP development unfolds in practice, with particular attention to tensions that emerge as stakeholders navigate competing goals, values and interests.

There is growing recognition that CPs are complex managerial interventions, shaped by internal and external contextual factors in healthcare organisations. These include social dynamics such as power relations, interests, leadership and motivation.^1 6 7^ The European Pathway Association framework^6^ highlights the impact of internal and external contexts on CP effectiveness, suggesting that context significantly influences CP development and CP outcomes. While the widely adopted seven-phase method by Vanhaecht *et al*^8^ and the eight-step method by Lodewijckx *et al*^9^ for CP development have proved valuable in the past 25 years, scholars increasingly advocate for a more systemic, holistic approach. Such an approach integrates patient experience, professional collaboration, contextual factors and organisational dynamics.^1^ In addition, CP development pursues multiple goals simultaneously—enhancing patient experience, integrating evidence-based practices and improving efficiency through standardisation and process optimisation—with the ultimate aim of improving team and patient outcomes.^1 4 10^ These goals are assumed to be complementary and mutually reinforcing.^14 10^,^13^ However, how these goals are prioritised and balanced in practice remains underexplored.

This recognition of contextual and goal-related complexity in CP development aligns with a growing body of research promoting the application of complexity thinking to healthcare (quality) improvement. Complexity is defined as ‘a dynamic and constantly emerging set of processes and objects that not only interact with each other, but come to be defined by those interactions’.^14^(p.42) This perspective challenges the prevailing linear thinking and emphasises the need to embrace unpredictability in improving health services.^15^,^17^ Scholars conceptualise healthcare organisations as complex adaptive systems, characterised by non-linear dynamics, distributed agency and context-sensitive learning.^15 18^

Contributing to this complexity is the presence of multiple, often conflicting values—such as autonomy, care quality and efficiency—that actors must navigate in quality improvement efforts.^19^,^24^ Greenhalgh *et al*^16^ capture this complexity with the concept of value complexity (or normative complexity^19^), defined as ‘the complexity arising from differences in people’s worldviews, interests and values, leading to mistrust, misunderstanding and conflict among stakeholders’.^16^(p648) In line with other scholars,^19 21 23 24^ this definition acknowledges that value is not fixed or predefined, but emergent and context-dependent—shaped through ongoing interactions, reflective deliberation among professionals, patients and managers. To support moral reasoning and reflection on value complexity, Greenhalgh *et al*^16^ propose 10 rules of thumb (see table 1), derived from diverse research traditions. These rules are not intended as prescriptive checklists, but as flexible, context-sensitive starting points for engaging with value complexity in empirical research. The rules encourage users to critically reflect on themes such as power dynamics, the use of numbers and visual representations, and uncertainty within complex moral landscapes.

**Table 1:** 10 rules of thumb on value complexity

Rule of thumb	Description
Partnership process is mission critical	Complex projects require trust-based partnerships with psychological safety for all. Focus should be on collective action rather than just deliverables as outputs.
Be alert to power dynamics	Power sharing in partnerships must consider subtle dynamics. Critical analysis can help reveal imbalances and ensure all voices are heard without reducing them to ‘empowered’ categories.
Engage with conflict positively	Conflict in value-complex projects is inevitable and can lead to deeper understanding. Conflict should be embraced rather than avoided to enhance collaboration and innovation.
Examine language carefully	Language shapes how issues are perceived and framed using the views of different actors. Understanding the rhetorical purpose and underlying assumptions and values can reveal different perspectives in partnerships.
Identify hidden value judgements in technology	Technologies often embed human judgements and values. Digital tools, for example, may marginalise certain users or perspectives. It is important to critique the social assumptions built into technologies.
Question numbers and metrics	The numbers and metrics used to define progress may reflect specific biases. Partnerships should question the origins and purposes of these measures to uncover hidden agendas or interests and the consequences of their use.
Encourage frame awareness	Different groups may see the world differently based on their institutional frames. Raising awareness of these frames helps build mutual understanding and cooperation despite differences.
Contemplate uncertainty	Uncertainty is inherent in real-world projects. Addressing it requires human qualities like humility, flexibility and courage, recognising that our understanding of complex issues is often limited.
Use deliberation for collective action	Scientific evidence alone will not determine action. Deliberation, which considers local constraints, values and priorities, helps guide real-world decisions. This process requires curiosity and respect for differing views.
Creative action cannot be scripted	Generalised knowledge and standardised interventions are insufficient to address real-world challenges. Action must be adaptive, involving experimentation (tinkering), and continuous data collection and analysis to find solutions.

Value complexity is relevant in CP development, where diverse perspectives must be negotiated and integrated—a challenge that our study seeks to address. In doing so, this study responds to Greenhalgh *et al*’s^16^ call for further reflection and empirical study of their rules of thumb, using them as a reflective lens in a CP development programme. The research question guiding this study was: ‘How does value complexity unfold in a CP development programme?’

## Methods

### Study settings and design

This study was a substudy of a 2-year action research (AR) programme on CP development, conducted in a Dutch specialised rehabilitation hospital (13 sites, ~800 employees and ~16 000 patients annually). The programme was initiated by the centre’s Executive Board and funded through a national AR grant scheme (ZonMw project number 05160062010008). A steering committee of senior management and medical leaders provided strategic oversight and alignment.

The AR programme aimed to support multidisciplinary teams in developing CPs that improved care quality, efficiency and innovation—including the integration of blended care (ie, the combination of face-to-face care with web-based digital interventions).^25^,^28^ In 3 CP development cycles, a total of 11 multidisciplinary teams were guided by an embedded research team in a quality collaborative approach, involving knowledge transfer, workshops and guided teamwork.^29^ Each multidisciplinary team was chaired by a leadership triad: a medical leader (rehabilitation physiatrist), a middle manager and a CP project leader (healthcare professional, eg, physiotherapist, speech therapist or social worker).

The research team consisted of three members and participated in the AR programme both as researchers and facilitators. The lead researcher coordinated the programme, coached the CP teams and organised the collaborative meetings. The research team facilitated expert-led knowledge exchange, interactive exercises and team-based learning throughout the collaborative meetings.

**Figure 1:**
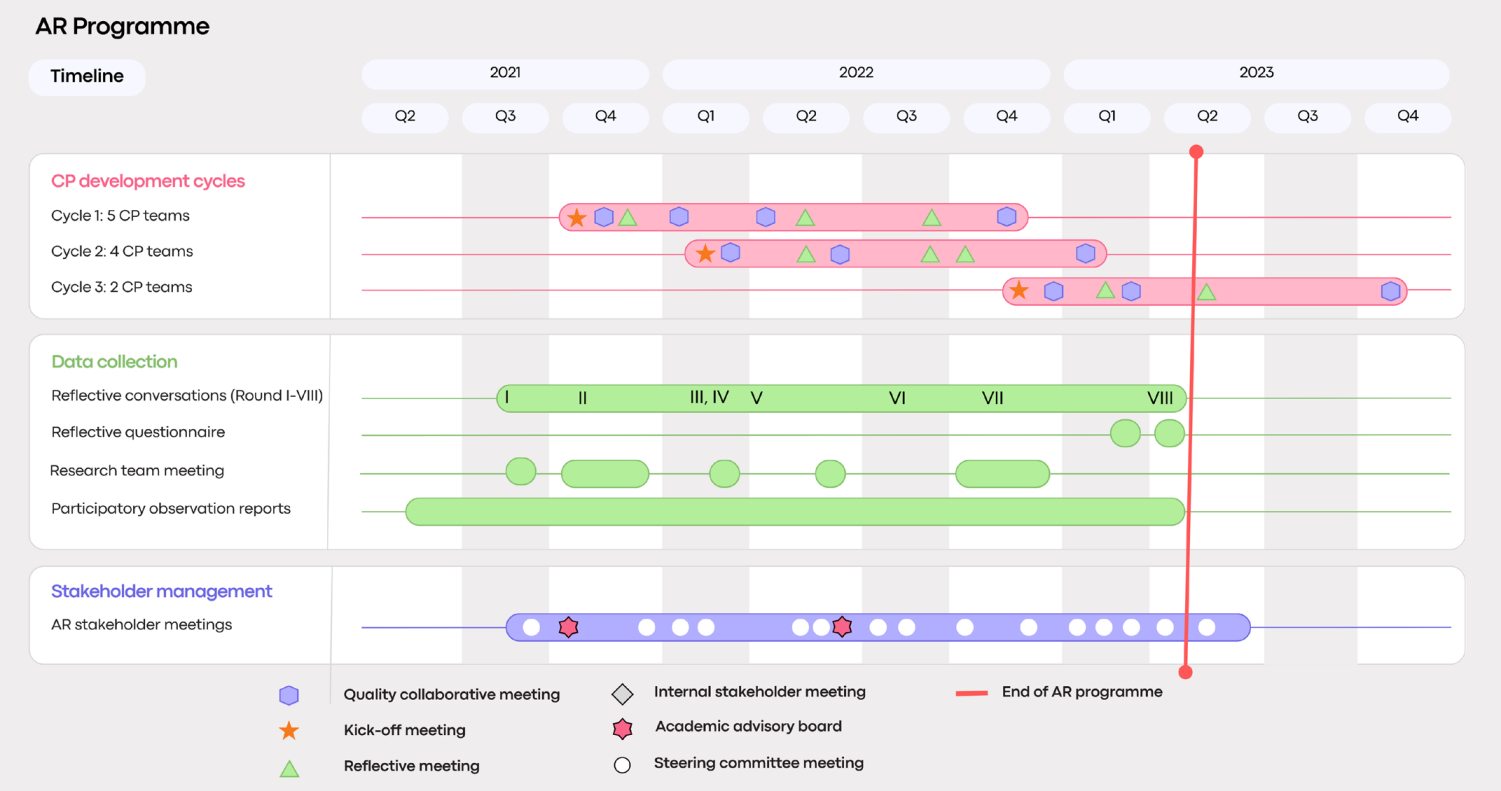
AR programme timeline. This timeline illustrates the longitudinal set-up of the AR programme and data collection rounds. Data analysis was conducted retrospectively, following a research-follow-action strategy. AR, action research; CP, care pathway.

This paper reports a qualitative single-case study within this broader AR programme, following an interpretivist paradigm.^30^,^32^ The substudy followed a ‘research-follow-action’ strategy, in which action and learning occurred during the CP programme phase, while systematic analysis took place retrospectively.^33^ This approach allowed the research team to stay close to practice during facilitation, while maintaining interpretive distance in the analytical phase. Figure 1 visualises the structure of the AR programme, including the three CP development cycles, the timing of data collection activities and AR stakeholder meetings (see figure 1).

### Data collection and participants

A multimethod data collection strategy was employed throughout the AR programme: (A) 169 participatory observation reports documented by the lead researcher, providing in-depth descriptions of complex situations in CP development; (B) a total of 44 reflective conversations conducted in eight rounds (rounds I–VIII) by the lead researcher to deepen insights; (C) two reflective questionnaires created in Microsoft Forms and distributed by email to gather additional perspectives and (D) 21 reflective research team meetings. An overview of the timing of the data collection is shown in figure 1.

Participants for the reflective conversations and questionnaires were selected through criterion sampling.^34^ A total of 56 professionals and managers were invited by email, of whom 44 accepted (79% participation rate; see table 2). These 44 reflective conversations were held with 26 unique participants, including representatives from all three roles in the leadership triad (N=21) as well as members of senior management (N=5). Each participant took part in one to four conversations, with an average length of 38 min. A topic guide was used to support the reflective conversations, allowing for flexibility and open-ended dialogue (see online supplemental appendix A). The first reflective questionnaire, distributed to leadership triad representatives (N=25), received 14 responses (56% response rate), while the second, sent to senior management (N=5), yielded 5 responses (100% response rate) (see table 3). The questionnaires consisted of open-ended questions designed to elicit reflective input (see online supplemental appendix B). Although participation and response rates varied, no systematic patterns of selective non-response were observed and the most important reason for not participating was ‘due to time constraints’. The reflective conversations and the research team meetings were audio-recorded and video-recorded using Microsoft Teams and transcribed verbatim. All data—including research team recordings, participatory observation reports, reflective conversation transcripts and questionnaire responses—were securely stored following General Data Protection Regulation (GDPR) guidelines.

**Table 2:** Invitations and participation in reflective conversations

				Breakdown			
Reflective conversation rounds	Invitations total (N)	Participants (N)	Participation rate (%)	CP middle manager (N)	CP medical leader (N)	CP project leader (N)	Senior manager (N)
I	5	4	80	4			
II	5	2	40			2	
III	4	4	100			4	
IV	11	11	100	3	4	4	
V	13	8	62	3	1	3	1
VI	9	7	78			7	
VII	1	1	100				1
VIII	8	7	88	3			4
	56	44	79%	13	5	20	6

CP, care pathway.

**Table 3:** Invitations and response to reflective questionnaires

				Breakdown			
Reflective questionnaire	Invitations total (N)	Response (N)	Response rate (%)	CP middle manager (N)	CP medical leader (N)	CP project leader (N)	Senior manager (N)
Questionnaire leadership triangles	25	14	56	2	4	8	
Questionnaire senior management	5	5	100				5
	30	19	63%	2	4	8	5

CP, care pathway.

### Data analysis

A thematic analysis was conducted retrospectively across all data gathered during the 2-year AR programme. While the broader AR programme involved iterative learning cycles, the analysis followed a ‘research-follow-action’ strategy, in which action and learning occurred during the programme, followed by systematic interpretation and synthesis after its completion.^33^

The thematic analysis was employed in accordance with Braun and Clarke’s^35^ six-phase methodology, blending inductive and deductive methods. In phase 1, the lead researcher familiarised herself with the participatory observation reports, identifying socially complex situations in CP development processes (eg, conflict, diverging perspectives or team demotivation) and the interventions deployed in response. These insights provided the foundation for generating the initial codes (phase 2). Next, the reflective conversations and questionnaires were inductively coded by the lead researcher using Atlas.ti V.24. All codes were reviewed and discussed in the research team to reach interpretive consensus.

In phase 3, the codes were grouped into four candidate themes. Conceptual maps were created in Atlas.ti to visualise thematic interconnections (see onlinesupplemental appendices C  D). The maps show how codes clustered into broader categories—such as financial viability, representation and consensus building—and how these related to each other. These visualisations facilitated the research team’s reflective dialogue and iterative refinement of the thematic structure. This reflexivity extended beyond individual researcher’s positioning to include reflection on how researcher perspectives shaped coding, theme development and meaning attribution.^36^

In phase 4, the research team consolidated the thematic structure. This process led to consensus on two overarching themes that reflected value complexity: goal alignment and decision-making in CP development. These were further analysed using value complexity as an interpretive lens.^16^ Drawing on elements of Greenhalgh *et al*’s^16^ rules of thumb, they were not applied prescriptively but used to explore how tensions around values, goal alignment and decision-making manifested in the data. This approach guided a reflective interpretation of the themes in relation to normative complexity. In phase 5, the inductive and deductive insights were balanced for theme definition and naming, combining and contrasting the themes and interventions. Finally, in phase 6, the two themes and interventions were described in full and presented in a narrative format.

### Research team and reflexivity

Reflexivity was an integral part of the methodological approach. Throughout the programme, the research team applied three-level reflexivity to critically examine their own assumptions and prevent ‘going native’.^3236^,^40^ This included: (1) the lead researcher maintaining a weekly reflexive journal, which served as a structured log of participatory observations used for both analytical and reflective purposes capturing field-level complexity and supporting critical reflection on role tensions, researcher impact and emergent value framings; (2) regular reflective meetings within the research team to critically interrogate their interpretations, power dynamics and interactions with participants and (3) two moments of academic reflection within an academic advisory board convened by the university. This board was chaired by a professor from an outsider stance and included the research team and rotating guests from the studied organisation and provided a space for critical dialogue and methodological reflection. These activities are represented in figure 1.

### Techniques to enhance trustworthiness

Techniques were applied to strengthen transparency, reliability and validity.^31^ To enhance transparency, the researchers created an audit trail documenting the research process. Reflexivity was practised throughout, enabling the research team to critically examine their own assumptions and to prevent ‘going native’.^38 39^ To strengthen reliability, detailed observations and conversation excerpts were provided to enable assessment of the transferability of the findings to other contexts and gaining a deeper understanding of the phenomena under study. The research team conducted intercoder reliability checks to ensure consistency in the coding and interpretation of data and refined the analysis through regular discussions, allowing diverse perspectives to shape the findings. Validity was enhanced by applying method triangulation, combining data from participatory observation reports, reflective conversations and questionnaires, as well as analysis triangulation combining inductive and deductive approaches to enhance depth and robustness in theme development.^41 42^ The Standards for Reporting Qualitative Research guideline led the writing process (see online supplemental appendix E).^43^

### Patient and public involvement

Patients and members of the public were not involved in the design, or conduct, or reporting, or dissemination plans of this study. This decision reflects the study’s focus on professionals’ perspective and their engagement in reflective dialogues on goal alignment and decision-making. However, in the broader AR programme in which this substudy was embedded, patients were actively involved as stakeholders in several CP development teams.^44^

## Results

The next section describes the overall approach of the CP development project. This is followed by an examination of value complexity in goal alignment and in decision-making and researchers’ interventions to engage with these complexities.

### Original goals and set-up

In the project’s initiation phase, all stakeholders (senior management, board and research team) agreed on a shared objective: employees would learn to develop CPs with smarter, more efficient processes to innovate care delivery and create uniformity across locations. At this stage, care quality, process improvement, innovation (including blended care) and financial viability were valued equally. A learning perspective was embedded through a quality collaborative setting, in which multidisciplinary teams were trained and guided in CP development. Each CP team was steered by a leadership triad, responsible for overseeing the development process and monitoring progress. The organisation was expected to gradually reduce its reliance on the researchers for guidance and project management, ultimately taking over full responsibility for the process.

### Value complexity in goal alignment

In our study, goal alignment refers to the process of ensuring that all stakeholders are aligned on shared objectives that balance care quality, financial viability and innovation. Shifting organisational priorities due to key staff changes and the resulting unfamiliarity with the project’s original goals—combined with a financial crisis in the first year—negatively affected goal alignment between senior management, CP leadership triangles, CP team members and the research team. After the project’s preparation, introduction and kick-off phases, organisational priorities shifted with an observable growth in managerial value for efficiency.

The first shift led management to increase pressure on the CP leadership triangles to develop financially viable CPs. This increased the power of managers, which negatively affected goal alignment in the leadership triangle. Consequently, the design process changed gradually from one driven by blended care content, patient centredness and process improvement to one driven by the need for a positive financial return. Management coined a metaphor for the situation: ‘How can you design a Rolls Royce when you only can afford a Lada?’ From the management perspective, only a good financial result was of value. For the CP team, reaching consensus on a redesigned CP implied agreeing to big changes to daily practices in terms of care content, use and deployment of digital resources and logistics. However, all the hard work this entailed for the CP team was considered secondary to ensuring a positive financial outcome. Management perceived the original design, based on the CP team’s vision of care, as leading to unrestrained, excessive and costly care provision. As management’s views no longer aligned with team members’, misunderstandings arose in leadership triangles and CP teams. This negatively affected the motivation to improve and innovate care processes for both patients and healthcare professionals. On the other hand, middle managers said that the opportunity to talk about balancing cost and care in the CP team was a significant benefit, as it reduced tension between management and care professionals and made the financial dilemmas easier to discuss.

Especially in our situation, when it became evident that we weren’t performing well financially, then yes, you must ensure that the care pathway will also yield benefits for [the organisation]. (Senior manager, Interview I_55)

… about envisioning the content, the feeling I got now was [that we were] being reined in by financial constraints. Yeah, I think that cost us time. And frustration. (Senior manager, Interview I_52)

In the second shift in response to the financial crisis, management focused on enhancing short-term care productivity to drive revenue generation. This emphasis on increased productivity influenced how management directed employees which, in turn, reduced the number of CPs developed. Despite formal approval and funding through an external subsidy, management limited the hours employees could spend on the CP project. This negatively affected the commitment of CP team members, who felt caught between two assignments: developing and implementing CPs and maintaining or increasing care productivity. This dual responsibility left them no time for project work which delayed the development process. As one CP project leader put it:

You feel the consequence of reducing the frequency of our meetings. We planned to alternate between meetings and working weeks, but that working time is gone. And now you feel like you’re not on the moving train anymore—you keep stepping on and off the platform, trying to catch up (CP project leader, I_40)

Team members at different levels echoed this tension. A coordinator described how shifting priorities eroded energy and a sense of shared commitment:

Patient care took priority, and after a while there just wasn’t much energy left. I really felt like I was standing alone. (CP project leader, I_41)

A medical leader reflected on how daily pressures overshadowed CP work and made participation difficult:

It’s incredibly busy, and there’s a lot of pressure to fill the gaps, especially for patient-related tasks. There are so many things to juggle, and this [mid-May] was the first available moment since early April to work on this—it took a month and a half. (Medical leader, I_38)

As the excerpt below illustrates, the noticeable decrease in CP development affected project outcomes and progress.

‘So, at first, everyone was quite enthusiastic about it. Like, okay, this is going to be a really great project. Then, as things progressed, [we] faced increasing pressure [to work] under the motto “all hands on deck”—I can’t recall the exact words. Well, you felt a bit embarrassed when you wrote down your hours for this. And that affected everyone.’ (Senior manager, Interview I_48)

In some CP teams, the misaligned goals touched on a pre-existing reluctance to work with either CPs or other innovations that challenge current practices and focus on the standardisation that interferes with professional autonomy. This reluctance, causing the core misalignment, noticeably affected the legitimacy of the project. As a result, some CP team members participated passively, and this sparked discussions about the organisational burden, feasibility and potential termination of the project. These effects then served as a catalyst in slowing down progress when the intrinsic motivation for developing the CP and adopting new working methods was already low.

To navigate value complexity related to goal alignment, the research team engaged CP teams in structured reflective dialogues that helped identify and address misalignments among stakeholders. These dialogues provided a platform for discussing the reduced motivation of CP teams, as well as the dilemmas and tensions the financial pressures created, giving stakeholders the space to explore key trade-offs. In these exchanges, stakeholders articulated their priorities and an expert-led training on the funding of specialised rehabilitation care was provided, fostering a shared understanding of the need to balance care quality, financial viability and operational efficiency. By promoting mutual understanding and collaboration across disciplines, the teams could work on a more cohesive CP vision. Also, the dialogues provided coping strategies for challenging times, enabling team members to collaboratively explore solutions. Preparatory sessions with leadership triangles clarified mutual expectations, further reinforcing alignment on project goals. However, the impact of the interventions varied across teams. While some teams successfully reached consensus and developed a shared vision despite the challenges, others struggled to overcome tensions and misunderstandings, further impeding CP development progress.

### Value complexity in decision-making

The significant delays observed in CP development (from design to experimentation to implementation) were partly due to slow decision-making. Decision-making involves the iterative process of making choices throughout CP development, particularly in designing and adapting CPs to reconcile varying practices and incorporate new clinical insights. There were four key aspects influencing this decision-making.

First, introducing the new form of leadership triangle revealed role uncertainty and differences in expectations. Since the triangle’s roles were new to the organisation, initial expectations regarding collaboration, role execution and task distribution were unstated and unaligned. This served as a catalyst for confusion and uncertainty, which clouded decision-making. Additionally, the triangle members felt inexperienced in their new roles. They had to learn to trust their collaborative partnership.

At first, it was a bit of a search, right? What and who and how? We did that together. What’s the right method, who’s responsible for what, how do we do it, and what can we expect? Lots of questions. (Middle manager, Interview I_34)

Second, the corporate culture followed the informal rule of pursuing consensus in decision-making among healthcare professionals. It was expected that CP team members had their colleagues’ mandate to compromise and make decisions. This mandate, however, was not always explicit or pre-agreed on. CP teams placed great importance on extensive consultation and careful alignment with other colleagues not on the CP team. As a result, the influence of the respective constituencies impacted the team members’ decision-making, leading to repeated discussions and re-evaluation of previously made choices in the attempt to reach consensus.

Now we’re back to the hot issues. I see us reaching a small consensus, speaking the same language. But then, in the team […] we often take one step forward and two steps back. Because [team members] consult their departments and think: Oh, no, this isn’t right. […] And that’s super hard. (CP project leader, Interview I_43)

We observed that a CP team’s shared vision of care positively contributed to their decision-making and ability to reach consensus. However, in team discussions, the members did not always mention their differences in perspective and motivation. Making the implicit explicit requires such skills as open questioning techniques, guiding reflective dialogues and creating a safe setting which stimulates openness. During the quality collaborative meetings, an action researcher facilitated the team dialogues. However, there was no professional dialogue guidance outside the meetings and the team’s own ability to guide these dialogues varied. Thus, decision-making was complicated by repetitive discussions and reconsidered decisions.

Third, the assumption was that all the disciplines involved in the care process were represented in the multidisciplinary CP team. However, when a CP spans multiple locations, choices were made in order to maintain a manageable team size. This implied that a physiotherapist of location A was expected to represent the physiotherapists of locations B and C. When CP teams were gathering input and proposition support, not having all stakeholders represented complicated their decision-making and their communication and coordination with the various settings. CP team members considered this a time-consuming and challenging task, especially when a team member did not work in or did not feel connected to a particular department or location. We observed team members making judgements of each other’s working methods as well as the pressure their respective constituencies exerted on them to preserve their preferred way of working. This negatively impacted the team, again delaying the decision-making process. However, teams that invested in building relationships outside the quality collaborative sessions experienced a positive response in their collaboration and decision-making process.

There was a huge culture of criticising the leadership and colleagues from other locations. […] There’s quite a bit of judgement on how you do it and how the other one does it. (Senior manager, Interview I_48)

Plus, there’s a bit of rivalry too. Yeah, that sounds a bit negative. You have to be careful… because then they think: oh, that location is doing well, we’re doing it wrong. (Middle manager, Interview I_54)

No, I think we got to know each other. I think that really made a difference because [CP X] is quite large with four separate locations… And now, because you also got to know them, just as people, as persons, how they think, their mindset, it was easier to work together. Yes, I think that really helped. (CP project leader, Interview I_45)

Fourth, the increased focus on designing financially viable CPs sparked discussions on balancing care costs and care quality. Team members found it hard to understand the care-related consequences of financially driven choices. Unfamiliarity with the ambiguous funding structure and uncertainty about the influence of the CP team on the financial outcome prolonged discussions and misunderstandings, which delayed the CP design process. Some teams developed CPs using evidence-based guidelines or national treatment frameworks. However, applying these recommendations sometimes implied a higher frequency or duration of therapy than was financially feasible under the existing reimbursement model. This discrepancy became apparent when applying the organisation’s calculation model, designed to assess the balance between care provision and expected reimbursement. As a result, tensions arose between clinically optimal care and financial viability. In such cases, CP teams had to weigh complex medical, ethical and organisational considerations, which made the decision-making process more challenging.

To navigate decision-making challenges, the research team introduced small-scale experimentation, allowing CP teams to test different pathway designs in practice. The CP teams found this approach helpful, appreciating the opportunity to learn from practical experience rather than hypothetical scenarios alone. Learning about each other’s working methods also reduced judgemental attitudes towards local practices, promoting a more collaborative approach to CP design. This hands-on experience fostered a culture of practical learning, encouraging CP teams to engage in meaningful and respectful discussions and supporting them in making well-considered CP design choices. To further enhance decision-making capacity, the teams received a cost-revenue calculation model that facilitated balanced decision-making on cost and care quality. This approach helped CP teams integrate financial considerations into their pathway designs and built their confidence in iterative decision-making. However, the effectiveness of these interventions differed between teams. Some teams made effective use of the tools and techniques, leading to constructive discussions and well-informed decisions, while others encountered difficulties in overcoming internal dynamics and structural challenges, resulting in decision-making delays.

## Discussion

This qualitative study explored value complexity in CP development processes, offering insights for practitioners (healthcare professionals, managers, CP project leaders) and (action) researchers involved in these complex processes. While the ‘rules of thumb’ by Greenhalgh *et al*^16^ have been cited in a limited number of peer-reviewed papers, this is, to our knowledge, the first empirical study using the rules in the context of CP development.^2245^,^48^

### Key findings and implications

The study identified goal alignment and decision-making as complex, multi-layered processes in CP development. In our study, the original project goals focused on developing blended CPs that balanced care quality, innovation and financial viability, while standardising practices across multiple locations and integrating digital tools. However, shifting organisational priorities, such as an emerging financial crisis, disrupted this balance. Management’s increased focus on financial outcomes over the patient-centred vision created tensions, hindering progress in both goal alignment and decision-making. While CP literature describes CP goals as complementary and assumes that goal alignment will naturally emerge over time to facilitate CP implementation, our findings show that this process is challenging.^7 49^ While core values—patient-centred care, efficiency and care-content improvement—remained constant, their prioritisation shifted throughout the CP development process, generating tension and hindering alignment at both management and CP team levels. Rather than being complementary or reinforcing each other, goals frequently competed, particularly under organisational pressure, leading to misunderstanding, tensions in collaboration and value conflicts.

The complexities of goal alignment and decision-making underscore the inherent tensions in CP development, reflecting the broader organisational challenge of balancing competing demands for innovation and efficiency. In decision-making processes, teams have to deal with competing goals and conflicting stakeholder interests.^45^ While the current CP literature provides limited guidance on how to achieve alignment or expedite decision-making processes amid competing priorities, our study showed mixed outcomes. Some CP teams navigated these tensions effectively, integrating conflicting stakeholder priorities and professional perspectives to maintain the coherence of their CPs, whereas others reached an impasse, halting progress. These challenges reflect organisational ambidexterity, which highlights the need to balance exploration (long-term innovation and risk-taking) and exploitation (short-term productivity and efficiency).^50^,^52^ In our study, exploitation became dominant during the financial crisis, as management prioritised immediate productivity gains over longer-term care innovations. This shift constrained CP teams’ ability to explore innovative approaches, illustrating the practical challenges of achieving ambidexterity in healthcare.

Our study highlights the dynamic, competing nature of CP goals, emphasising the importance of periodic reassessment to support iterative goal alignment. Our findings align with the literature on adaptive evaluation in complex health improvement initiatives, which underlines the value of formative evaluation in navigating competing priorities.^53^,^55^ Formative evaluation continuously creates real-time feedback loops, enabling early identification of challenges, proactive misalignment management and sustained collaboration.^53^,^55^ This aligns with complexity science insights, which stress flexibility, reflexivity and adaptability in navigating multistakeholder initiatives in dynamic contexts.^15^,^17^ Embedding formative evaluation in CP development strengthens the teams’ adaptive decision-making and resilience in managing organisational pressures.

The key findings and their implications described above suggest that decision-making and goal alignment in CP development is not only a technical or procedural activity, but also a moral process shaped by divergent values and interests. In some teams, shared moral ground was actively cultivated through dialogue and iterative experimentation, allowing actors to align sufficiently to constructively make decisions. In other teams, however, alignment on values and interests became constrained due to strategic positioning or lack of managerial authority. This was particularly evident when financial pressure increased, which impeded decision-making. Value convergence requires active facilitation; it cannot be assumed to emerge naturally. This aligns with normative complexity, which refers to the coexistence of conflicting but legitimate values, such as patient-centredness, efficiency and professional autonomy. These conflicting values complicate or even obstruct efforts at value convergence.^16 19 23^ Rather than seeking full alignment, decision-making in such contexts requires recognition of moral tensions and facilitation of dialogical spaces where diverse perspectives can be expressed, questioned and negotiated. In this view, value conflicts are not problems to be resolved, but realities to be surfaced and engaged with.

### A reflexive use of Greenhalgh *et al*’s rules of thumb in CP development

Greenhalgh *et al*’s^16^ rules of thumb (see table 1) were used as a reflexive lens to interpret how value tensions emerged around goal alignment and decision-making in CP development. This interpretive stance supported exploration of the moral and relational dimensions of these processes in a context-sensitive manner, consistent with the intended purpose of the rules. While this approach provided valuable insights, it also revealed challenges in their application. Value complexity hindered consensus building and mandate acquisition when diverse stakeholder views were considered. In our study, partnerships were strained and power dynamics became prominent, especially under increased pressure and during tense discussions, particularly when stakeholders held conflicting views. For example, power dynamics between management and CP teams intensified during financial crises, aligning with the second rule regarding recognising power relations. Similarly, efforts to build connections, trust and mutual understanding, as outlined in the first rule, were crucial for facilitating decision-making and effectively communicating with respective constituencies. However, these efforts were not always sufficient to overcome existing tensions.

Our study also highlights the influence of language and framing in goal realignment, consistent with the fourth and seventh rules. The ‘Lada metaphor’ used by management exemplified how framing could reinforce financial pressures, further complicating goal alignment. Teams that managed to establish partnerships and trust, central to the first rule, were better equipped to navigate these challenges and achieve consensus. Despite these efforts, achieving consensus and acquiring mandates remained difficult, particularly when divergent stakeholder priorities persisted.

While this study’s findings support the utility of these rules, we concur with Greenhalgh *et al*^16^ that they lack predictive power. Translating the rules into a framework or checklist would detract from their purpose and the essence of complexity science and adding more rules would not be beneficial. Also, the rules are subject to multiple interpretations and require a strong theoretical knowledge base in social science and humanities (eg, on power differentials, framing) to understand and differentiate between rules (eg, between the fourth and seventh rules). Furthermore, the rules interact, as demonstrated by the interplay of power (second rule) and conflict (fourth rule) in partnerships (first rule). Despite these challenges, our findings underscore their usefulness for exploring value complexity in complex processes such as CP development.

Action researchers, who aim to bridge practice and science by applying scientific knowledge to practice and developing interventions to support desired change processes, are encouraged to use the rules of thumb as a reflexive lens in complex change processes. Grounded in rich theoretical insights, these rules provide guidance in ongoing problem analysis. This aligns with the concept of ‘value exnovation’ proposed by Wehrens *et al*,^23^ which emphasises the action researchers’ role in uncovering implicit, often conflicting values in healthcare practices. In our study, such exnovation became evident in the reflexive dialogues as these dialogues revealed implicit assumptions about what ‘should’ matter in CP development, particularly the tension between financial efficiency and patient-centred care. These moments of reflection helped teams to explore new strategies to balance competing priorities. Recent research by Oldenhof *et al* identifies three strategies for engaging with value complexity: working around it (eg, through temporary fixes), working against it (by reinforcing dominant value logics), and working with value complexity—for example, through inclusive dialogue and reflexive spaces that surface tensions constructively.^22^ Our findings align with scholars who advocate for complexity-informed project approaches that embed incrementalism, reflexivity and adaptive learning.^15^,^17^[^56^] In this light, our study contributes to the enrichment of CP development methodologies enabling teams to navigate value complexity more effectively. It does so by: (1) demonstrating how ongoing reflection and evaluation enhances adaptability and teams’ learning capacity and (2) emphasising the importance of deliberately selecting strategies that ‘work with’ value complexity, as proposed by Oldenhof *et al*.^22^ Future research is needed to explore how the rules of thumb can be used across diverse healthcare settings and to further develop practical strategies that support reflexivity, value exnovation and ‘working with’ value complexity in complex change processes.

### Strengths and limitations

To the best of our knowledge, this is the first empirical study on value complexity in CP development using Greenhalgh *et al*’s^16^ rules of thumb. This 2-year longitudinal AR on CP development provides a robust method for understanding value complexity over time, because the natural embedding of action researchers in the organisation enhanced the credibility of the findings. The research team’s ongoing reflexivity was another strength, as they critically examined their biases and assumptions to mitigate the risk of ‘going native’.^34^ The study also has certain limitations. As this is a single case study in one (multilocation) specialised rehabilitation hospital, transferability to other contexts may be constrained. Both the single-case nature and the specialised rehabilitation setting may have shaped the observed dynamics, as organisational culture is known to influence team functioning in such contexts.^57 58^ To counter this effect, the researchers provide rich, detailed observations and interview excerpts to give other researchers the opportunity to assess the transferability of the findings.^30 31^ Additionally, the strategy chosen for criterion sampling might have led to selection bias, especially as multiple interviews were conducted with the same respondents over time. This may have resulted in an over-representation of coordinators’ perspectives, as they were often more consistently available for participation. We encourage further research on value complexity both in and beyond CP development in other (types of) healthcare organisations. Moreover, although patients were involved in the AR programme, they were not included in the data collection or analysis of this substudy. As patient experience is a key dimension of value in healthcare, future studies should explore how patients perceive and contribute to goal alignment and decision-making. Finally, while CP teams were encouraged to incorporate evidence-based care, our study did not examine how perceived differences in evidence strength across disciplines influenced CP development. Future research could explore how such disciplinary variations affect value negotiation and decision-making.

## Conclusions

This study explored value complexity in CP development by using Greenhalgh *et al*’s^16^ rules of thumb. It illustrates the dynamic processes of goal alignment and decision-making that arise from conflicting interests, shifting priorities and evolving organisational contexts, underscoring the importance of reflexivity, dialogue and trust-building among stakeholders in effectively navigating these conflicting goals. The findings highlight that achieving and maintaining goal alignment is vital for successful CP development and requires ongoing management and formative evaluation. While the rules of thumb are valuable for understanding and addressing value complexity, Greenhalgh *et al*’s^16^ work builds on rich theory and so using the rules necessitates a solid theoretical foundation and an awareness of the interplay between the different rules.

This study advocates for a complexity-informed approach that enriches CP development methodologies with continuous reflection and adaptability, equipping healthcare professionals and researchers to respond effectively to complex challenges while promoting successful collaborative practices among diverse stakeholders. The findings demonstrate the added value of using the rules of thumb, providing action researchers with diagnostic, analytical and reflective guidance to navigate the inherent challenges of CP development effectively.

We encourage further research on value complexity both in and beyond CP development in (other types of) healthcare organisations. This includes exploring how reflective approaches—such as Greenhalgh *et al*’s rules of thumb—can be strengthened and translated into practical strategies to enhance reflexivity and dialogue among stakeholders to further support effective decision-making and goal alignment in CP development.

## Supplementary material

10.1136/bmjopen-2024-098157 online supplemental file 1

10.1136/bmjopen-2024-098157 online supplemental file 2

10.1136/bmjopen-2024-098157 online supplemental file 3

10.1136/bmjopen-2024-098157 online supplemental file 4

10.1136/bmjopen-2024-098157 online supplemental file 5

## Data Availability

No data are available.
