# Supplementary material for: Navigating value complexity in care pathway development: a qualitative case study
Source: BMJ Open. 2025 Aug 13;15(8):e098157. doi: 10.1136/bmjopen-2024-098157 (PMC12352197; doi:10.1136/bmjopen-2024-098157)
Supplement: online supplemental file 1 [file bmjopen-15-8-s001.pdf]

CONVERSATION GUIDE REFLECTIVE CONVERSATIONS

ROUND I

Dutch version

Motivatie en keuze voor het zorgpad

1. Wat gaf voor jou of jullie team de doorslag om voor dit zorgpad te kiezen?
2. Welke behoeften of kansen zagen jullie in deze doelgroep of dit zorgpad?
3. Wat hoop(te) je hiermee te bereiken, voor patiënten en/of de organisatie?

Samenwerking en teamvorming

4. Hoe is de samenstelling van het zorgpadteam tot stand gekomen?
5. Wie neemt binnen het team welke verantwoordelijkheden op zich (zorginhoud, bedrijfsvoering, projectleiding)?
6. Hoe kijk je aan tegen het betrekken van ervaringsdeskundigen of patiënten bij dit traject?

Randvoorwaarden voor implementatie en borging

7. Wat is er volgens jou nodig om het zorgpad goed te implementeren én structureel te borgen?
8. Wat zijn eerdere lessen of ervaringen met borging van zorgvernieuwing in jouw praktijk of organisatie?
9. Hoe kijk je aan tegen monitoring, evaluatie en eventuele bijstelling van het zorgpad?

Waarde en stuurinformatie

10. Hoe geef je invulling aan waardegedreven zorg binnen dit pad (kwaliteit, kosten, patiëntervaring)?
11. Wat zie jij als de rol van data, klinische uitkomstmaten, IT en financiën in dit traject?

Leiderschap en projectaanpak

12. Wat vraagt dit traject van jouw rol?
13. Wat is jouw leiderschapsstijl en hoe wil je je betrokkenheid vormgeven?
14. Hoe zie je de rol van een team facilitator, en welke competenties zijn daarvoor nodig?

Zorg voor motivatie, draagvlak en brede implementatie

15. Hoe zorg je ervoor dat collega's mee blijven doen en gemotiveerd blijven?
16. Wat is er nodig om dit zorgpad breder gedragen en toegepast te krijgen in de praktijk?

English version

Motivation and rationale for selecting the care pathway

1. What prompted you or your team to choose this particular care pathway?
2. What needs or opportunities did you identify within this patient group or care process?
3. What did you hope to achieve — for patients and/or the organisation — by developing this pathway?

Collaboration and team formation

4. How was the care pathway team constituted?
5. How are responsibilities within the team distributed (e.g. clinical leadership, operational coordination, team facilitator)?

6. What are your thoughts on involving patient representatives or experiential experts in this process?

Conditions for implementation and sustainability

7. In your view, what is needed to implement and sustain the care pathway effectively?
8. What lessons have you learned from previous experiences with embedding care innovation in practice?
9. How do you approach the monitoring, evaluation, and potential adjustment of care pathways over time?

Value and data-driven management

10. How do you apply value-based healthcare principles within this care pathway (e.g. outcomes, costs, patient experience)?
11. What role do data, clinical metrics, IT systems, and financial insights play in this trajectory?

Leadership and project organisation

12. What does this project require from you in your role?
13. How would you describe your leadership style and preferred level of involvement?
14. What is your understanding of the role of a team facilitator, and what qualities are important in fulfilling that role?

Sustaining motivation and broad uptake

15. How do you ensure that colleagues remain engaged and motivated?
16. What do you think is needed to support wider adoption and consistent application of the care pathway in practice?

ROUND II

Dutch version

Motivatie en keuze voor het zorgpad /

1. Wat gaf voor jou of jullie team de doorslag om voor dit zorgpad te kiezen?
2. Welke behoeften of kansen zagen jullie in deze doelgroep of dit zorgpad?
3. Wat hoop(te) je hiermee te bereiken — voor patiënten en/of de organisatie?

Samenstelling en rolverdeling in het team

4. Hoe is de samenstelling van het zorgpadteam tot stand gekomen?
5. Hoe worden verantwoordelijkheden binnen het team verdeeld (zorginhoud, bedrijfsvoering, team facilitator)?
6. Wat zijn jouw overwegingen bij het betrekken van ervaringsdeskundigen of patiënten?

Implementatie en borging

7. Wat is er volgens jou nodig om het zorgpad goed te implementeren én structureel te borgen?
8. Welke lessen heb je geleerd uit eerdere implementatie- of borgingspogingen?
9. Hoe kijk je aan tegen monitoring, evaluatie en eventuele bijstelling van zorgpaden?

Waarde en informatiegestuurd werken

10. Hoe geef je invulling aan waardegedreven zorg binnen dit pad (kwaliteit, kosten, patiëntervaring)?
11. Welke rol spelen data, klinimetrie, IT en financiën in dit traject?

Rol en leiderschap

12. Wat vraagt dit traject van jou in jouw rol (bv. als manager, coördinator, professional)?
13. Hoe zou je jouw leiderschapsstijl omschrijven?

Betrokkenheid en motivatie

14. Hoe zorg je ervoor dat collega's betrokken blijven en gemotiveerd zijn?
15. Wat is er nodig om bredere toepassing en inbedding van het zorgpad te realiseren?

Autonomie versus standaardisatie

16. Hoe kijk je aan tegen de spanningen tussen autonomie van zorgprofessionals en standaardisatie via zorgpaden?

English version

Motivation and rationale for selecting the care pathway

1. What prompted you or your team to choose this particular care pathway?
2. What needs or opportunities did you identify in this patient group or care process?
3. What did you hope to achieve — for patients and/or the organisation?

Team composition and roles

4. How was the care pathway team assembled?
5. How are responsibilities distributed within the team (clinical leadership, operational coordination, facilitator)?
6. What are your considerations around involving experiential experts or patient representatives?

Implementation and sustainability

7. What do you think is needed for effective implementation and long-term sustainability of the pathway?
8. What lessons have you learned from earlier attempts at implementing or embedding changes?
9. How do you approach monitoring, evaluation, and adjustment of care pathways?

Value-based and data-informed working

10. How do you apply value-based healthcare principles within this pathway (quality, costs, patient experience)?
11. What role do data, clinical metrics, IT and finance play in this process?

Professional roles and leadership

12. What does this project require from you in your professional role (e.g. as a manager, coordinator, or clinician)?
13. How would you describe your leadership style?

Engagement and motivation

14. How do you ensure continued engagement and motivation among colleagues?
15. What is needed to support wider adoption and embedding of the care pathway?

Autonomy versus standardisation

16. How do you view the tension between professional autonomy and standardisation through care pathways?

ROUND III

Dutch version

1. Hoe heb je de startbijeenkomst ervaren, en wat heeft het je opgeleverd of juist niet?
2. Hoe kijk je aan tegen jouw rol als zorgpadteam projectleider en de samenwerking in de driehoek?
3. Wat valt je op in de dynamiek binnen het team of tussen locaties?
4. Welke voorwaarden zijn belangrijk om je rol goed te kunnen vervullen en voortgang te boeken?
5. Wat verwacht je van het vervolgtraject, en waar zie je kansen of knelpunten?
6. Hoe kijk je aan tegen de besluitvorming binnen het traject?
7. Wat is volgens jou de rol en meerwaarde van ervaringsdeskundigen in het ontwerpproces?
8. Waar zie je de meeste vernieuwing of impact voor de patiënt of het team?
9. Welke ondersteuning heb je nodig om je rol goed te vervullen?

English version

1. How did you experience the kick-off session, and what did you gain out of it?
2. How do you view your role as team facilitator and the collaboration within the leadership triad team?
3. What stands out to you in the team dynamics or between different locations?
4. What conditions are essential to fulfil your role and make progress?
5. What are your expectations for the next phase, and where do you see opportunities or bottlenecks?
6. How do you view the decision-making process within this trajectory?
7. What do you see as the role and added value of involving service users in the design process?
8. Where do you expect the greatest innovation or impact — for patients or the team?
9. What support do you need to effectively carry out your role?

ROUND IV

Dutch version

Voorbereiding en verwachtingen

1. Welke verwachting had je voorafgaand aan de bijeenkomst?
2. Hoe heb je je voorbereid op de werkconferentie?

Groepsdynamiek en samenwerking

3. Hoe verliep de samenwerking in jouw groep?
4. Wat viel je op in de groepsdynamiek of onderlinge rollen?

Werkvormen en procesopbouw

5. Hoe heb je het werken met de droom en fases ervaren?
6. Hoe werkte de mindmap- of brainstormsessie voor jullie groep?

Opbrengsten en inzichten

7. Wat heb je geleerd of als waardevol ervaren tijdens de werkconferentie?
8. Waar zag je mogelijke vernieuwing of verandering ontstaan?

Vervolg en borging

9. Wat is er nodig om vervolgstappen te zetten in het team of traject?
10. Hoe zie jij het vervolg of de borging van dit proces voor je?

Reflectie algemeen

11. Is er verder nog iets dat je wilt delen of kwijt wilt?

English version

Preparation and expectations

1. What were your expectations beforehand?
2. How did you prepare for the work conference?

Group dynamics and collaboration

3. How did the collaboration within your group go?
4. What stood out to you in the group dynamics or roles?

Interactive exercises and process structure

5. How did you experience working with the shared vision and phases?
6. How did the mind map or brainstorming session benefit your group?

Outcomes and insights

7. What did you learn or find valuable during the work conference?
8. Where did you observe potential innovation or change emerging?

Follow-up and sustainability

9. What is needed to take the next steps within your team or project?
10. How do you envision the follow-up or embedding of this process?

General reflection

11. Is there anything else you would like to share?

ROUND V

Dutch version

Terugblik en leerervaringen

1. Hoe kijk je terug op de afgelopen maanden in het traject?
2. Wat heb je zien gebeuren in je team of organisatie?
3. Wat zijn volgens jou belangrijke leerpunten uit deze periode?

Ervaring werkconferentie en inhoudelijke thema's

4. Hoe heb je de derde werkconferentie ervaren?
5. Wat vond je van de inhoudelijke thema's zoals sturen op waarde, indicatoren, teamdynamiek?
6. Welke onderdelen vond je inspirerend of juist lastig toepasbaar?

Timing en fasering

7. Hoe kijk je aan tegen het moment waarop bepaalde onderwerpen zijn geïntroduceerd?
8. Had je sommige thema's liever op een ander moment besproken?

Samenwerking en rollen

9. Hoe kijk je aan tegen de rolverdeling binnen jullie team en de samenwerking met de manager en medisch leider?

10. Wat is belangrijk in de samenwerking binnen de leiderschapsdriehoek?
11. Wat heb je geleerd over jouw rol als team facilitator?

Vervolg en behoefte aan ondersteuning

12. Wat is er nodig om de volgende stap te zetten met jullie zorgpad?
13. Wat helpt jou en het team om tot concrete output te komen?
14. Welke ondersteuning of structuur zou helpend zijn?

English version

Reflection and lessons learned

1. How do you look back on the past months of the programme?
2. What developments have you seen within your team or organisation?
3. What do you see as key lessons learned?

Experience of the work conference and thematic content

4. How did you experience the third work conference?
5. What was your impression of the thematic content such as value-based performance monitoring, indicators, and team dynamics?
6. Which elements did you find inspiring or difficult to apply?

Timing and sequencing

7. How do you experience the timing of the introduction of certain topics
8. Would you have preferred to discuss some themes at a different moment during the development process?

Collaboration and roles

9. How do you view the division of roles in your team and the collaboration with the manager and medical leader?
10. What is important for collaboration within the leadership triad?
11. What have you learned about your role as a team facilitator?

Next steps and support needs

12. What is needed to take the next step with your care pathway?
13. What helps you and the team to produce concrete output?
14. What kind of support or structure would be helpful?

ROUND VI

Dutch version

Terugblik en geleerde lessen

1. Hoe kijk je terug op het traject tot nu toe?
2. Wat zijn volgens jou belangrijke lessen of inzichten uit deze periode?
3. Wat heeft voor jullie goed gewerkt, en wat niet?
4. Welke invloed had de groepsdynamiek of samenwerking tussen locaties?

Ervaring met bijeenkomsten en inhoudelijke thema's

5. Wat vond je van de inhoud en timing van de bijeenkomsten?
6. Wat heb je gehad aan onderdelen zoals indicatoren, samen beslissen of blended care?
7. Welke elementen vond je moeilijk toepasbaar?

Ontwikkeling zorgpad en implementatie

8. Wat is er veranderd in het zorgpad en waarom?
9. Hoe is het implementatieproces verlopen en waar loop je tegenaan?
10. Waar zie je inhoudelijke winst of meerwaarde?

Persoonlijke rol en ondersteuning

11. Hoe ervaar je jouw rol als zorgpadteam projectleider of teamlid?
12. Wat heb je nodig om deze rol goed te vervullen?
13. Hoe heb je de begeleiding of coaching vanuit het project ervaren?

Vooruitblik en vervolgstappen

14. Wat is er nodig om komende periode stappen te zetten?
15. Wat hoop je dat de volgende bijeenkomst oplevert?
16. Hoe kunnen we de voortgang borgen of verbeteren?

English version

Reflection and lessons learned

1. How do you evaluate the programme so far?
2. What are key lessons or insights from this period?
3. What worked well for your team, and what did not?
4. What role did group dynamics or collaboration across locations play?

Experience with meetings and thematic content

5. What was your impression of the content and timing of the meetings?
6. What did you gain from elements such as indicators, shared decision-making, or blended care?
7. Which components were hard to apply in practice?

Care pathway development and implementation

8. What changes were made in the care pathway and why?
9. How has the implementation process gone and what challenges have you faced?
10. Where do you see added value or improvement in the new approach?

Personal role and support

11. How do you experience your role as a team lead or participant?
12. What do you need to fulfil this role effectively?
13. How have you experienced the coaching or support from the project team?

Looking ahead and next steps

14. What is needed to make progress in the coming months?
15. What do you hope the next session will bring?
16. How can progress be safeguarded or strengthened?

ROUND VII

Dutch version

Dynamiek en betrokkenheid

1. Hoe kijk je terug op de voorbereiding, aanwezigheid en betrokkenheid van de leiderschapsdriehoeken?
2. Wat valt je op in de manier waarop managers hun rol vervullen binnen het zorgpadtraject?
3. Wat zijn mogelijke oorzaken voor afhaken of beperkte betrokkenheid?

Tijdsbesteding en realistische planning

4. Hoe kijk je aan tegen de afgesproken tijdsinvestering (bijv. 2 uur/week voor 6 teamleden)?
5. Wat blijkt in de praktijk haalbaar en wat niet?
6. Hoe kunnen we teams helpen om ritme en voortgang te behouden?

Draagvlak en organisatiekeuzes

7. Hoe ervaren teams de ruimte die ze formeel hebben gekregen vanuit de organisatie?
8. Welke signalen spreken elkaar tegen (bijv. formele goedkeuring vs. gevoelde ruimte)?
9. Wat is er nodig om dit traject structureel te borgen binnen de organisatie?

Alternatieve werkwijzen

10. Welke werkwijzen (bijv. langere sessies met minder frequentie) zouden efficiënter kunnen zijn?
11. Hoe kunnen teams eigen keuzes maken in hun werkritme binnen de kaders van het programma?

English version

Team dynamics and engagement

1. How do you reflect on the preparation, attendance, and engagement of the leadership triad(s)?
2. What do you observe in the way managers fulfil their role within the care pathway programme?
3. What might explain limited engagement or withdrawal?

Time investment and realistic planning

4. How do you view the agreed time commitment (e.g. 2 hours/week for 6 team members)?
5. What proves feasible in practice, and what not?
6. How can we support teams in maintaining momentum and a steady rhythm?

Support and organisational alignment

7. How do teams experience the organisational space formally given to them?
8. What signals appear contradictory (e.g. formal approval vs. experienced constraints)?
9. What is needed to embed this programme structurally within the organisation?

Alternative working methods

10. What alternative working methods (e.g. longer sessions less frequently) might be more effective?
11. How can teams shape their own work rhythm within the programme framework?

Round VIII

Dutch version

Terugblik en verwachtingen

1. Hoe kijk je terug op de start en je verwachtingen bij aanvang van het project?
2. Welke verwachtingen had je bij de samenwerking met het projectteam?

Verandering en fasering

3. Zie je bepaalde fases of periodes terug in hoe het project zich ontwikkelde?
4. Wat veranderde er in jouw rol of betrokkenheid door de tijd heen?

Leerervaringen en verrassing

5. Werd je tijdens het traject verrast - positief of negatief?
6. Wat heb je zelf geleerd of zien veranderen bij anderen?

Rollen en samenwerking

7. Hoe kijk je aan tegen de rolverdeling tussen managers, coördinatoren en therapeuten?
8. Wat is er nodig voor goede samenwerking binnen en tussen teams?

Herkenbare patronen binnen de organisatie

9. Wat zag je gebeuren dat je zou typeren als typisch voor deze organisatie?
10. Wat gebeurde er juist dat daarvan afweek?

Krachten en belemmeringen

11. Wat heeft het project versterkt of juist vertraagd?
12. Welke factoren maakten het lastig om voortgang te boeken?

Impact van uitspraken of frames

13. Welke uitspraken of overtuigingen zijn bepalend geweest voor de koers van het project?
14. Welke frames hebben het proces ondersteund of juist tegengewerkt?

English version

Reflections and expectations

1. How do you evaluate on the project's start and your expectations at the time?
2. What were your expectations regarding collaboration with the project team?

Change and phases

3. Do you recognise certain phases or stages in how the project evolved?
4. What changed in your role or level of engagement over time?

Learning experiences and surprises

5. Were you surprised during the project - in a positive or negative way?
6. What did you learn yourself, or observe changing in others?

Roles and collaboration

7. How do you evaluate the division of roles between managers, coordinators, and therapists?
8. What is needed for effective collaboration within and across teams?

Recurring organisational patterns

9. What did you observe that felt typical for this organisation?
10. What stood out as different from the usual practice?

Enablers and barriers

11. What helped to accelerate or hinder the project?
12. Which factors made it difficult to maintain progress?

Impact of statements or frames

13. Which statements or beliefs shaped the course of the project?
14. What frames supported or hindered the process?
