# Supplementary material for: Navigating value complexity in care pathway development: a qualitative case study
Source: BMJ Open. 2025 Aug 13;15(8):e098157. doi: 10.1136/bmjopen-2024-098157 (PMC12352197; doi:10.1136/bmjopen-2024-098157)
Supplement: online supplemental file 2 [file bmjopen-15-8-s002.pdf]

REFLECTIVE QUESTIONNAIRE QUESTIONS

RO_06 20230328

Dutch version

1. Wat heb je / hebben jullie nodig om de doorontwikkeling van het zorgpad gaande te houden?
2. Wat is naar jouw idee nodig om als persoon en als team de motivatie te houden hiervoor?
3. Wat heeft in het verleden naar jouw idee goed of juist niet goed gewerkt als het gaat om het doorzetten van de beweging die in gang is gezet?
4. Kun je een voorbeeld noemen van een verandering of project dat is afgemaakt? En hoe is dat toen aangepakt? Wat maakte dat het werkte?
5. Is er nog iets dat je zou willen meegeven aan de groep van 28 maart?

English version

1. What do you (or your team) need to sustain the ongoing development of the care pathway?
2. In your view, what is needed to maintain motivation — both individually and collectively — to continue this work?
3. Reflecting on the process; what has facilitated or hindered the sustained momentum of the initiated changes?
4. Can you give an example of a successfully completed change or project How was it approached, and what factors or actors contributed to its success?
5. Is there anything you would like to share with the group meeting on 28 March?

RO_04B_20230417

Dutch version

1. Hoe kijk jij terug op het project van afgelopen twee jaar en hoe de opdracht is opgepakt?
2. Hoe kijk jij terug op de samenwerking met de universiteit?
3. Hoe heb jij de inzet van MV als projectleider en actieonderzoeker ervaren?
4. Wat ik verder nog kwijt wil, is...

English version

1. Looking back, how do you reflect on the project over the past two years and the way the assignment was taken up?
2. How do you evaluate the collaboration with the university?
3. How did you experience MV’s role as project leader and action researcher?
4. Is there anything else you would like to add or share?
