# Supplementary figures and images for: Navigating value complexity in care pathway development: a qualitative case study
Source: BMJ Open. 2025 Aug 13;15(8):e098157. doi: 10.1136/bmjopen-2024-098157 (PMC12352197; doi:10.1136/bmjopen-2024-098157)

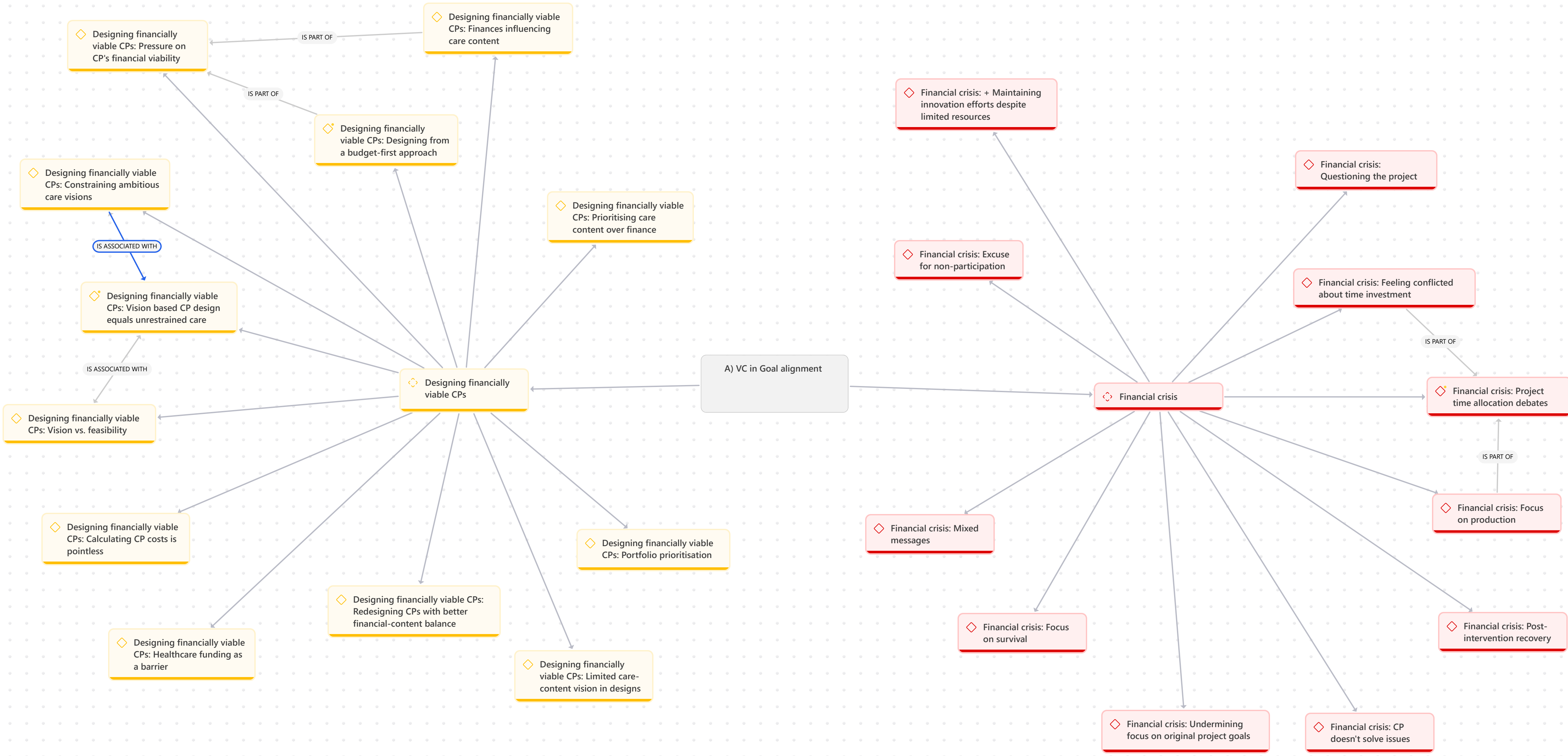

Supplement: online supplemental file 3 [file bmjopen-15-8-s003.pdf]

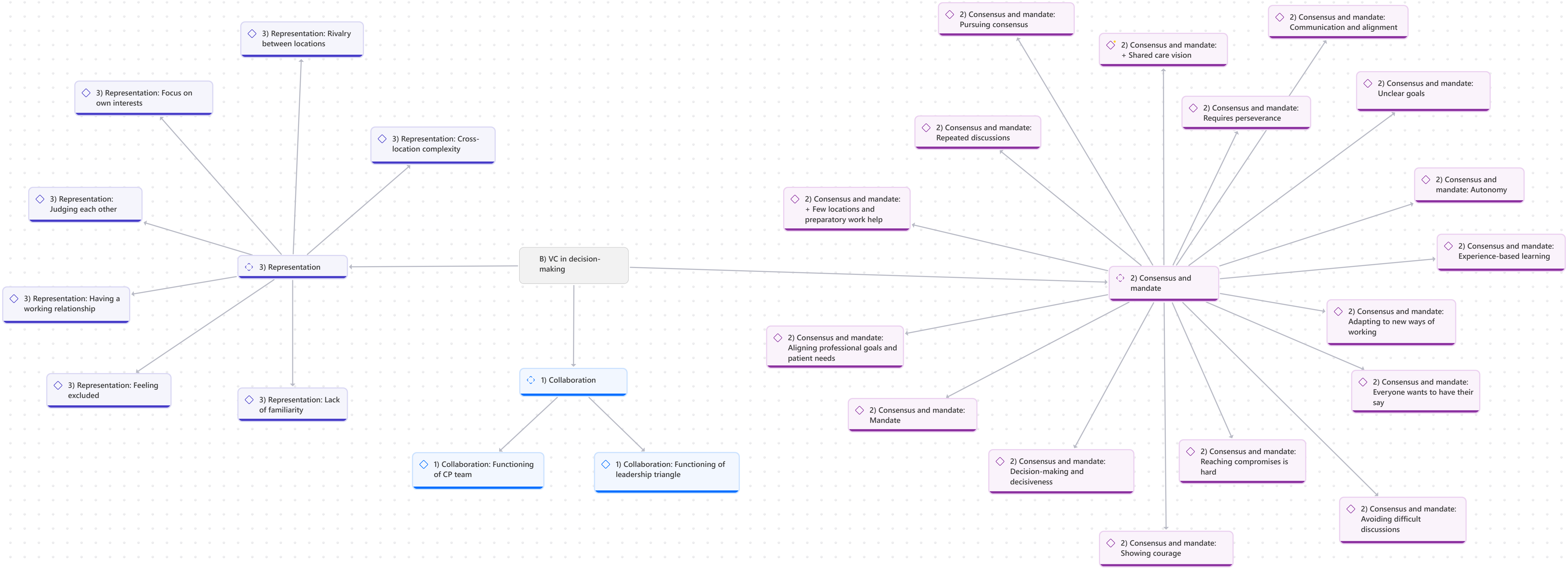

Supplement: online supplemental file 4 [file bmjopen-15-8-s004.pdf]
