# Supplementary material for: Navigating value complexity in care pathway development: a qualitative case study
Source: BMJ Open. 2025 Aug 13;15(8):e098157. doi: 10.1136/bmjopen-2024-098157 (PMC12352197; doi:10.1136/bmjopen-2024-098157)
Supplement: online supplemental file 5 [file bmjopen-15-8-s005.pdf]

SRQR Checklist Manuscript

SRQR Item	Description	Response
S1. Title	Concise description of the nature and topic of the study, identifying it as qualitative	The title, "Navigating Value Complexity in Care Pathway Development: A Qualitative Case Study," clearly indicates the qualitative nature of the research.
S2. Abstract	Summary of key elements of the study	The abstract provides a comprehensive summary, including background, purpose, methods, main findings, and conclusions (p. 2 of the manuscript).
S3. Problem Formulation	Description and significance of the problem/phenomenon studied; review of relevant theory and empirical work; problem statement	The introduction outlines the complexity of care pathway (CP) development and emphasises the importance of understanding value complexity (pp. 5-7).
S4. Purpose or Research Question	Purpose of the study and specific objectives or questions	The study aims to empirically explore value complexity in CP development and reflect on the application of Greenhalgh et al.'s rules of thumb (p. 7).
S5. Qualitative Approach and Research Paradigm	Approach used and guiding theory	A qualitative single case study was conducted in an action research framework, following an interpretivist paradigm (p. 8).
S6. Researcher Characteristics and Reflexivity	Characteristics of the researchers that may influence the research, and their relationship with participants	Reflexivity was expanded to include participatory observation reports and regular discussions to mitigate bias. Thorough efforts were made to manage the dual roles of researchers as facilitators, including self-reflection practices to prevent bias and 'going native' (p. 8).
S7. Context	Setting/site and salient contextual factors	Conducted in a Dutch medical rehabilitation centre, the study provides detailed contextual information (p. 8).
S8. Sampling Strategy	How participants were selected and the criteria for sampling	Added details on sampling rationale, including why 26 participants were chosen. Criterion sampling was used to select leadership triangles, higher management, and committee members (pp. 8-9).
S9. Ethical Issues Pertaining to Human Subjects	Ethical approval, participant consent, and confidentiality measures	Ethical approval was granted by the Erasmus University Ethical Review Committee, and GDPR compliance was ensured (p. 10).
S10. Data Collection Methods	Types and details of data collection procedures	Data were collected through reflective conversations, questionnaires, and participatory observation reports. Procedures, including recording methods are described in detail (pp. 8-9).

SRQR Item	Description	Response
S11. Data Collection Instruments and Technologies	Instruments used and any changes during the study	MS Teams was used for recording reflective conversations, and MS Forms for administering questionnaires. No significant changes to instruments were made (p.8- 9).
S12. Units of Study	Number and relevant characteristics of participants	The study involved 26 reflective conversation participants, including leadership triangles, managers, and steering committee members, and 19 reflective questionnaire participants, described in the participant section (p. 8-9).
S13. Data Processing	Methods for processing data, including transcription and anonymisation	Reflexive conversations were transcribed verbatim and pseudonymised. Data were securely stored following GDPR guidelines (p. 9-10).
S14. Data Analysis	How themes or patterns were identified and developed	A thematic analysis was conducted using Braun and Clarke's methodology, combining inductive and deductive approaches (p. 9).
S15. Techniques to Enhance Trustworthiness	Strategies like triangulation and audit trails	Specific reflexive practices, including triangulation across data sources, audit trails, and inter-coder reliability checks, were added to support consistency in data interpretation (p. 10).
S16. Synthesis and Interpretation	Main findings and their interpretation	The findings are presented in narrative form, identifying themes related to goal alignment and decision-making in CP development (p. 10-18).
S17. Links to Empirical Data	Evidence to support findings, such as quotes or excerpts	The manuscript includes extensive participant quotes and examples to substantiate findings (p. 10-18).
S18. Integration with Prior Work, Implications, Transferability, and Contributions to the Field	Connection to existing literature or theoretical frameworks	The discussion integrates findings with complexity science literature and Greenhalgh et al.'s theoretical framework (p. 18-23).
S19. Limitations	Trustworthiness and limitations of findings	Expanded discussion on limitations of the single-case study design, mentioning steps taken to support transferability through rich contextual details, enhancing applicability to other settings (p. 22).
S20. Conflicts of Interest	Potential sources of influence or perceived influence on the research	The authors have no conflicts of interest to declare (p. 23).
S21. Funding	Sources of funding and the role of funders	Funded by ZonMw; the funding body had no role in the study design, data collection, or reporting (p. 3).
