# Supplementary material for: Navigating value complexity in care pathway development: a qualitative case study
Source: BMJ Open. 2025 Aug 13;15(8):e098157. doi: 10.1136/bmjopen-2024-098157 (PMC12352197; doi:10.1136/bmjopen-2024-098157)
Supplement: Supplementary file 6 [file bmjopen-2024-098157.reviewer_comments.pdf]

PEER REVIEW HISTORY

BMJ Open publishes all reviews undertaken for accepted manuscripts. Reviewers are asked to complete a checklist review form and are provided with free text boxes to elaborate on their assessment. These free text comments are reproduced below.

ARTICLE DETAILS

Title (Provisional)

Navigating Value Complexity in Care Pathway Development: A Qualitative Case Study

Authors

Visser, Mildred; de Mul, Marleen; Ahaus, Kees; Weggelaar-Jansen, Anne Marie JWM

VERSION 1 - REVIEW

**Reviewer**	**1**
**Name**	**van Harten, Wim**
**Affiliation**	**University of Twente, Department of Health Technology and Services Research**
**Date**	**31-Dec-2024**
**COI**	**None**

This paper reports on an action research project related to Care Pathway (CP) development in a medical rehabilitation centre in The Netherlands using the Greenhalgh et.al. rules of thumb on value complexity.

In all this can be an interesting case analysis on CP development in a complex environment, though the setting seems rather specific and the structure of project and analysis are not easily accessible to the international reader.

In the introduction the concept of CPs is well explained, whereas the concept of "value" in CP development is not. One would expect an equally reflective passage on value. Further a choice is made to use Greenhalghs rules of thumb, whereas there are many more frameworks in this regard, Value based Health care not the least. There is no argumentation, why this frame work is the most suitable or preferred against other options. It is also stated that "CP development OFTEN involves social challenges etc..." whereas my experience is that that is only a minority of cases or depending on a specific setting or case.

The setting is described as " a medical rehabilitation centre". I take that it is a specialized rehabilitation hospital (according to Dutch regulations a "specialized hospital") ; internationally the chosen phrasing could also mean a geriatric rehabilitation hospital, geriatric rehabilitation or alike. This also relates to the culture as factor as described under "corporate culture". The culture that is described seems especially related to multidisciplinary specialist rehabilitation,

which may have a less decisive nature than for instance in oncology and more decisive culture than pediatrics. Also the extend of the team, who is involved, and are these also interviewed or participating in the discussions are relevant aspects? This earns a more thorough description of the setting, environment and processes than is now apparent from the paper.

In all the structure can be much improved. The action research project is not described a priori, but rather ex post, whereas it is interesting for the reader to learn what was planned, in what sequence and timing, and what was actually realized. Maybe in a table or time line?

Further there is mention of phases, shifts and decision making, whereas I would expect the rules of thumb to be followed as structure for both the results and discussion. Especially as this project might shed some innovative light on this very recent (2023) work. Also this could be visualized in a graph or table, especially translating the rules into experiences of this project.

Some where it is mentioned that the action research is a 2 year project, but it is not clear whether the funding also related to supporting the teams in their proces and how the "ownership" of the project was embedded in the center.

The set of stake holders, and which of those were actually involved in the project is not clear. Further in the methods an extensive description is given on a consensus process, that is not traceable in a very structured way in the results.It seems as if 8 steps (phases?, conversations) are reported upon, but it is not clear whether that leads to an actual consensus or conclusion, whether that was uniform or diverse etc. etc.In between data and methods there are two passages on Thrustworthiness and Value complexity reporting on issues that impress as experiences during the action research process. In relation to earlier remarks my advise would be to use this information to either describe the setting (when expected a priori) or to present them as results (when experienced ex post). Organisational issue are always relevant in action research and it is not uncommon that financial issues play a role; it would be helpful to better distinguish the development, the research and managerial roles in the earlier project and action research descriptions.

Later on in the paper there seems to be an emphasis on introducing "blended care" in the CPs. That is an issue worth investigating as such as it is known to be complex and brings its own issues. Whether or not this was the case should be clear form the beginning (as part of the project description?) and rather deserves some specific background literature.

The lack of structure or use of the rules to structure, the results, makes the conclusions and discussion difficult to follow. And advising the alike application of the rules of thumb without looking at alternatives is challenging.

In all a restructuring of the paper would improve the readability, logic's and generalizability. The discussion part can than be properly reviewed.

It is not clear why the patients perspective is not taken into account, as there is a lot of literature on patient involvement in CP development. This should especially be relevant in rehabilitation.

Lastly style can be improved, sometimes the authors use very long sentences (e.g. 123-127) and there some typo´s.

**Reviewer**	**2**
**Name**	**Gartner, Jean-Baptiste**
**Affiliation**	**Université Laval, Département de management**
**Date**	**18-Jan-2025**
**COI**	**None**

Comments to the author(s)
This qualitative study tries to "empirically explore value complexity by applying the Greenhalgh et al."

INTRODUCTION/LITERATURE REVIEW
- The theme is extremely relevant and interesting, and the very comprehensive literature on the concept of care pathways demonstrates a detailed understanding of the field's knowledge. However, you could expand on your thoughts and your introduction to the concept of value in healthcare. Here are a few suggestions that might strengthen your introduction:

- Sturmberg, J. P. (Ed.). (2016). The value of systems and complexity sciences for healthcare (No. 8801). Switzerland: Springer International Publishing.
- Sturmberg, J. P. (2018). Health system redesign. How to make health care person-centered, equitable, and sustainable. Cham: Springer.
-

- Formulation of the research question: as an interpretivist paradigm methodology, it seems counterproductive to 'limit' yourselves to the normative framework of Greenhalgh et al. In my opinion, the research question should have been more open, leaving space for other themes to emerge; and runs counter to systems thinking and the science of complexity (which leaves room for emergence, in particular)

METHODS
The methodology section is well-constructed, but certain points need to be strengthened:

Study design
The design of the study seems rather appropriate and is in line with the research question.

Research team and reflexivity
The reflexivity of researchers is a key point of credibility in qualitative research. The methods used should be better described and based on the methodological literature on qualitative research. See for example:

- Denzin, N. K., & Lincoln, Y. S. (Eds.). (2011). The Sage handbook of qualitative research. sage

Data collection and sampling
The material mobilised seems rich and dense. A table or figure representing the material would be relevant. In addition, in order to reflect the evolution of the project over 2 years and the dynamic aspect of the controversies, the table/figure should show the chronology of the information gathered.

Data analysis
The analysis and coding process is very well explained (particularly with regard to techniques to enhance trustworthiness). However, you could add a word about coding to the paragraph on data analysis. In this paragraph, it is not clear whether the coding was carried out by at least two double-blind researchers or more. Nevertheless, the method seems very convincing.

Minor points: I don't think 'Patient and public involvement' is in the right place.

RESULTS
Overall, the results are very interesting. But at this stage, the proposition still needs to be strengthened to convince the reader.

- The shift in value linked to the quality of care towards the economic approach to value is a classic in healthcare improvement project. At this stage, the demonstration needs to rely even more on the hardware. For example, the verbatims are mainly (if not exclusively) from senior managers. Yet it is the perspective of middle managers and care professionnals that should be presented to support the existence of the controversy (and remove the feeling that this controversy is primarily felt by the researchers themselves, reflected, for example, in the phrase : “This negatively affected the commitment of CP team members, who felt caught between two assignments: developing and implementing CPs”).
- The part about decision-making is fundamental for me. That is to say, can the moral values of the actors converge to make a constructive decision for the CP (and its actors), or can they not converge and so it is a question of power games and domination in decision-making.
- From my point of view, the patient's view of what is valuable is the missing angle in the proposal.

DISCUSSION
The discussion is particularly relevant and some references (in particular Wehrens et al.) could have enriched your introduction (which remains too limited on value and complexity).

The limitations are clear and well presented.

Minor points: I

CONCLUSION
The conclusion is sufficient and interesting. I remain convinced that the Greenhalgh et al. framework should not be used as a normative framework but as a reflexive framework (which is what you say, by the way). This is therefore a second research perspective: to develop a framework of moral values in healthcare or to strengthen the one of Greenhalgh et al.

REFERENCES

- BMJ open guidelines recommend to put DOI in the format : “doi: 10.xxxxxx” for all articles.

IN BRIEF:

The overall objective has certainly been achieved. Overall, the manuscript is already of good quality and well written. Like any manuscript, it could be improved by reviewing the literature in the introduction section, improving the methodology section by producing a figure showing the data materials, reinforcing in the results section the role of the material in supporting the inferences made, in particular by cross-referencing the perspectives of the different categories of players involved. The results are overall relevant and interesting.

Good luck for the next steps.

**Reviewer**	**3**
**Name**	**Tao, Wenjuan**
**Affiliation**	**West China Hospital of Sichuan University**
**Date**	**04-Jun-2025**
**COI**	**None**

General Comments

This qualitative single-case study explores value complexity in CP development by applying Greenhalgh et al.'s "rules of thumb" framework in a Dutch medical rehabilitation center over a two-year action research program. The study identifies goal (mis)alignment and prolonged decision-making as key themes affecting CP development and offers insights into navigating value complexity in healthcare improvement initiatives.

Major Comments

1.The use of Greenhalgh et al.'s rules of thumb is a key strength, as it provides a structured lens to explore value complexity. However, the manuscript could better articulate how each rule was applied in the analysis. For instance, while the discussion references rules such as power dynamics (Rule 2) and language framing (Rule 4), the specific analytical process linking these rules to the data is somewhat unclear. Consider including a brief subsection in the Methods or Results section to clearly explain how each rule was operationalized during data analysis, such as indicating which codes or themes corresponded to specific rules. This would enhance transparency and replicability for readers unfamiliar with the rules.

2.The single-case study design is noted as a limitation, which is appropriate, but the manuscript could do more to support transferability to other healthcare settings. While rich descriptions are provided, the discussion could explicitly address how the findings might apply to other contexts (e.g., acute care, primary care, or other rehabilitation settings).

Minor Comments

1."Value complexity" is central to this study, but the introduction only provides a definition of "complexity". I am still confused about what "value complexity" actually means.

2.The code networks in Appendices A and B are difficult to read and interpret. The manuscript would benefit from a brief description of what these code networks entail (e.g., key themes or relationships visualized).

3.While the study offers valuable insights, the practical guidance remains somewhat abstract. The interventions described (reflexive dialogues, small-scale experimentation) need more detailed implementation guidance. What specific facilitation techniques were most effective? How might organizations without embedded action researchers implement these approaches?

4.Table 2 & 3: Response rates vary considerably across cycles (50%-100%). Was there any pattern to non-participation that might introduce bias?

VERSION 1 - AUTHOR RESPONSE

Reviewer 1: Prof. dr. Wim van Harten

Thank you for your detailed and constructive review. Your feedback helped us improve the conceptual clarity, contextual transparency, and methodological rigour of our manuscript. We appreciate your critical questions about structure, stakeholder engagement, and the use of Greenhalgh et al.'s rules of thumb. Please find our point-by-point responses below.

This paper reports on an action research project related to Care Pathway (CP) development in a medical rehabilitation centre in The Netherlands using the Greenhalgh et.al. rules of thumb on value complexity.

In all this can be an interesting case analysis on CP development in a complex environment, though the setting seems rather specific and the structure of project and analysis are not easily accessible to the international reader.

Thank you for acknowledging the potential of our paper and your feedback.

In the introduction the concept of CPs is well explained, whereas the concept of "value" in CP development is not. One would expect an equally reflective passage on value. Further a choice is made to use Greenhalghs rules of thumb, whereas there are many more frameworks in this regard, Value based Health care not the least. There is no argumentation, why this frame work is the most suitable or preferred against other options.

Thank you for pointing out the need for more conceptual depth regarding the notion of 'value' in relation to complexity in CP development. In the revised introduction, we now elaborate on the meaning of value in healthcare from a complexity-informed perspective, drawing on recent literature that frames value as emergent, negotiated, and context-dependent. Rather than framing value through the lens of Value-Based Healthcare (VBHC), we adopt a sociological and complexity-informed perspective. This perspective aligns with our interpretivist approach and the study's focus on stakeholder interaction and moral reasoning. We also explain our rationale for choosing the Greenhalgh et al.'s rules of thumb. Rather than presenting them as the only available framework, we now introduce it more clearly as a reflexive tool. Furthermore, we incorporated recent scholarship in both the introduction and the discussion

INTRODUCTION: […] In parallel, a growing body of research promotes the application of complexity thinking to healthcare (quality) improvement. Complexity is defined as 'a dynamic and constantly emerging set of processes and objects that not only interact with each other, but come to be defined by those interactions'.[1 p.42] This perspective challenges the prevailing linear thinking and emphasises the need to embrace unpredictability in improving health services.[2-4] Scholars conceptualise healthcare organisations as complex adaptive systems, characterised by non-linear dynamics, distributed agency, and context-sensitive learning.[2,5]

Contributing to this complexity is the presence of multiple, often conflicting values – such as autonomy, care quality, and efficiency - that actors must navigate in quality improvement efforts.[6-11] Greenhalgh et al.[3] capture this complexity with the concept of value complexity (or normative complexity[6]), defined as 'the complexity arising from differences in people's worldviews, interests, and values, leading to mistrust, misunderstanding, and conflict among stakeholders'.[3 p648] In line with other scholars[6,8,10,11], this definition acknowledges that value is not fixed or predefined, but emergent and context-dependent - shaped through ongoing interactions, reflective deliberation among professionals, patients, and managers. To support moral reasoning and reflection on value complexity, Greenhalgh et al.[3] propose ten rules of thumb (see Table 1), derived from diverse research traditions. These rules aren't intended as prescriptive checklists, but as flexible, context-sensitive starting points for engaging with value complexity in empirical research. The rules encourage users to critically reflect on themes as power dynamics,

the use of numbers and visual representations, and uncertainty within complex moral landscapes.

It is also stated that "CP development OFTEN involves social challenges etc..." whereas my experience is that that is only a minority of cases or depending on a specific setting or case.

Thank you for this observation. We appreciate your perspective and recognise that experiences with CP development may vary across settings and healthcare systems. In our manuscript, the phrasing 'often involves social challenges' was intended to reflect insights from the previous paragraphs describing CP development as a complex process, shaped not only by clinical and organisational factors but also by social dynamics such as conflicting interests and power dynamics. We have rephrased the sentence to stay closer to the framing used in the introduction

INTRODUCTION: […] Against this background, this study explores how CP development unfolds in practice, with particular attention to tensions that emerge as stakeholders navigate competing goals, values, and interests. [..]

Value complexity is relevant in CP development, where diverse perspectives must be negotiated and integrated - a challenge that our study seeks to address. In doing so, this study responds to Greenhalgh et al.'s[3] call for further reflection and empirical study of their rules of thumb, using them as a reflexive lens in a CP development programme. The research question guiding this study was: "How does value complexity unfold in a CP development programme?"

The setting is described as "a medical rehabilitation centre". I take that it is a specialized rehabilitation hospital (according to Dutch regulations a "specialized hospital") ; internationally the chosen phrasing could also mean a geriatric rehabilitation hospital, geriatric rehabilitation or alike.

Thank you for pointing this out. We agree that the original phrasing may be ambiguous in an international context. In the revised Study settings section, we now clarify that the setting was a Dutch specialised medical rehabilitation hospital.

STUDY SETTINGS: This study was a sub-study of a two-year action research (AR) programme on CP development in a Dutch specialised rehabilitation hospital (16 sites, ~800 employees, ~16,000 patients annually).

This also relates to the culture as factor as described under "corporate culture". The culture that is described seems especially related to multidisciplinary specialist rehabilitation, which may have a less decisive nature than for instance in oncology and more decisive culture than paediatrics.

Thank you for this insightful observation. We agree that team culture may vary considerably between care domains such as oncology, rehabilitation, and paediatrics, and that such differences may influence decision-making and collaboration in CP development. In response, we clarified in the manuscript that the study took place in a Dutch specialised medical rehabilitation hospital, where multidisciplinary collaboration and shared decision-making are embedded in daily practice. We also added a reflection in the 'Strengths and limitations' section, noting that the observed consensus-oriented dynamics may reflect cultural features specific to this setting. This is supported by earlier research showing how organisational culture shapes rehabilitation team functioning.[12,13]

STRENGTHS AND LIMITATIONS: As this is a single case study in one (multi-location) specialised rehabilitation hospital, transferability to other contexts may be constrained. Both the single-case nature and the specialised rehabilitation setting may have shaped the observed dynamics, as organisational culture is known to influence team functioning in such contexts.[12,13] To counter this effect, the researchers provide rich, detailed observations and interview excerpts to give other researchers the opportunity to assess the transferability of the findings.[14,15]

Also the extend of the team, who is involved, and are these also interviewed or participating in the discussions are relevant aspects? This earns a more thorough description of the setting, environment and processes than is now apparent from the paper. In all the structure can be much improved. The action research project is not described a priori, but rather ex post, whereas it is interesting for the reader to learn what was planned, in what sequence and timing, and what was actually realized. Maybe in a table or time line?

Thank you for this valuable multi-faceted comment. The study primarily reports on an empirical sub-study. We aim to publish a paper in the nearby future explaining the action research more thoroughly. In response, we have taken several steps to improve clarity and transparency on the action research in this paper. First, we revised the Study settings and design section to include a clearer description of the two-year action research (AR) programme, the role and composition of the embedded research team, and the broader engagement of organisational stakeholders. We also added Figure 1, which visualises the longitudinal structure of the action research programme. The figure presents the three CP development cycles, key moments of data collection, and the evolving structure of stakeholder engagement over time. This timeline also provides insight into the intended sequencing of activities. Second, we expanded the Data collection and participants section to specify that participants in the reflexive dialogues and questionnaires included representatives from the roles in the leadership triad, as well as senior managers. Lastly, we revised the research team and reflexivity section to describe how reflexivity was applied to enhance analytical rigour, while information about the team's embedded role in the AR programme is now integrated in Study settings and design. We hope these adjustments enhance the structural coherence and contextual transparency of the manuscript.

STUDY SETTING AND DESIGN: […] The programme was initiated by the centre's executive board and funded through a national AR grant scheme (ZonMw project number 05160062010008). A steering committee of senior management and medical leaders provided strategic oversight and alignment.

The AR programme aimed to support multidisciplinary teams in developing CPs that improved care quality, efficiency, and innovation - including the integration of blended care (i.e. the combination of face-to-face care with web-based digital interventions).[16- 19] In three CP development cycles, a total of eleven multidisciplinary teams were guided by an embedded research team in a quality collaborative approach[20], involving knowledge transfer, workshops and guided teamwork. Each multidisciplinary team was chaired by a leadership triad: a medical leader (rehabilitation physiatrist), a middle manager, and a CP project leader (healthcare professional, e.g., physiotherapist, speech therapist, or social worker).

The research team consisted of three members, and participated in the AR programme both as researchers and facilitators. The lead researcher coordinated the programme, coached the CP teams, and organised the collaborative meetings. The research team facilitated expert-led

knowledge exchange, interactive exercises, and team-based learning throughout the collaborative meetings.

This paper reports a qualitative single-case study within this broader AR programme, following an interpretivist paradigm.[15,21,22] The sub-study followed a 'research- follow-action' strategy[23], in which action and learning occurred during the CP programme phase, while systematic analysis took place retrospectively. This approach allowed the research team to stay close to practice during facilitation, while maintaining interpretive distance in the analytical phase. Figure 1 visualises the structure of the AR programme, including the three CP development cycles, the timing of data collection activities and AR stakeholder meetings (see Figure 1).

Figure 1: AR programme timeline. This timeline illustrates the longitudinal set-up of the AR programme and data collection rounds. Data analysis was conducted retrospectively, following a research-follow-action strategy.

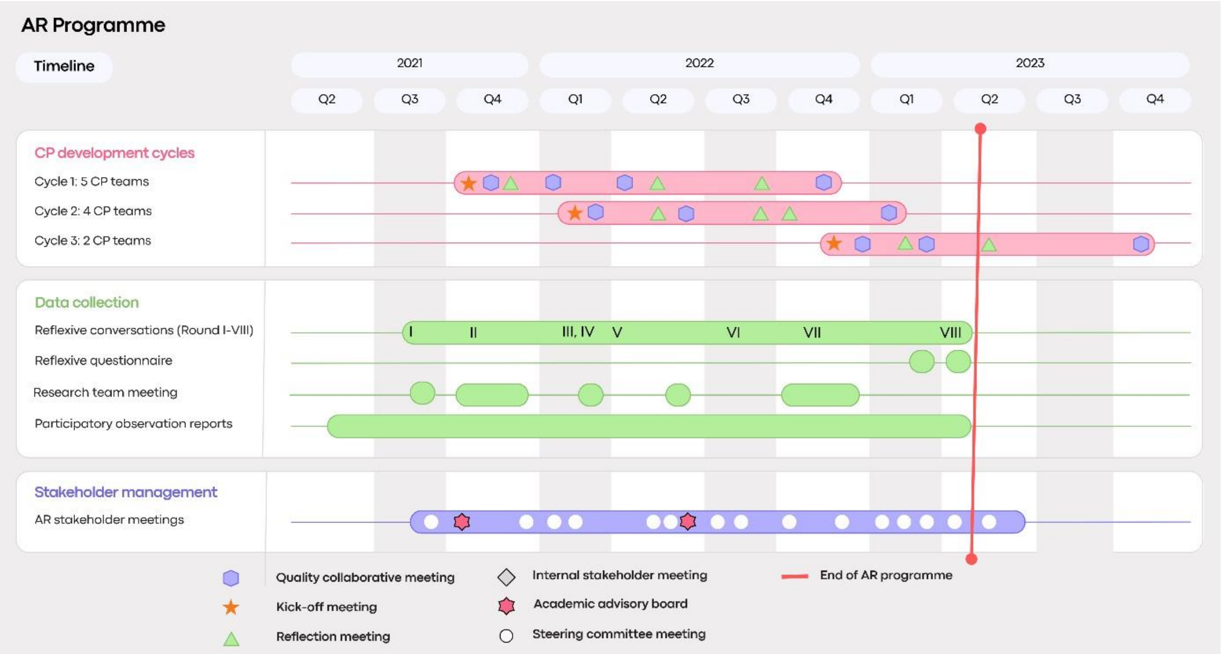

DATA COLLECTION AND PARTICIPANTS: A multi-method data collection strategy was employed throughout the AR programme: A) 169 participatory observation reports documented by the lead researcher, providing in-depth descriptions of complex situations in CP development; B) a total of 44 reflexive conversations conducted in eight rounds (Rounds I-VIII) by the lead researcher to deepen insights; C) 2 reflexive questionnaires created in Microsoft Forms and distributed by email to gather additional perspectives, and D) 21 reflexive research team meetings. An overview of the timing of the data collection is shown in Figure 1.

Participants for the reflexive conversations and questionnaires were selected through criterion sampling.[30] A total of 56 professionals and managers were invited by email, of whom 44 accepted (79% participation rate; see Table 2). The sample included representatives from all three roles in the leadership triad (N=21) as well as members of senior management (N=5). Each participant took part in one to four conversations, with an average length of 38 minutes. The first reflexive questionnaire, distributed to leadership triad representatives (N=25), received 14 responses (56% response rate), while the second, sent to senior management (N=5), yielded five responses (100% response rate) (see Table 3).Although participation and response rates varied, no systematic patterns of selective non-response were observed and the most important reason for not participating was "due to time constraints". The reflexive conversations and the research team meetings were audio- and video-recorded using Microsoft Teams and transcribed verbatim. All data – including research team recordings, participatory observation reports, reflexive conversation transcripts, and questionnaire responses – were securely stored in the Erasmus University's Blackberry Workspace.

RESEARCH TEAM AND REFLEXIVITY: Reflexivity was an integral part of the methodological approach. Throughout the programme, the research team applied three- level reflexivity 23,25] to critically examine their own assumptions and prevent 'going native'.[26-28] This included: (1) the lead researcher maintaining a weekly reflexive journal, which served as a structured log of participatory observations used for both

analytical and reflexive purposes capturing field-level complexity and supporting critical reflection on role tensions, researcher impact, and emergent value framings; (2) regular reflexive meetings within the research team to critically interrogate their interpretations, power dynamics, and interactions with participants, and (3) two moments of academic reflection within an academic advisory board convened by the University. This board was chaired by a professor from an outsider stance, and included the research team and rotating guests from the studied organisation, and provided a space for critical dialogue and methodological reflection. These activities are represented in Figure 1.

Further there is mention of phases, shifts and decision making, whereas I would expect the rules of thumb to be followed as structure for both the results and discussion. Especially as this project might shed some innovative light on this very recent (2023) work. Also this could be visualized in a graph or table, especially translating the rules into experiences of this project.

We understand your expectation that the rules of thumb by Greenhalgh et al. could be used to structure both the results and the discussion. However, in our study the rules were used as a reflexive lens during the later stages of analysis. The rules helped us to deepen our interpretation of how value tensions around goal alignment, and decision-making manifested in the data. Rather than applying the rules prescriptively or as data collection categories, we drew on them to guide normative reflection and sense-making. This interpretive and reflexive stance aligns with Greenhalgh et al.'s intent:

"We sought to present this theory not as fixed predictive statements but as a set of heuristics or rules of thumb—that is, points of departure for reflection and situated moral reasoning ("thinking through" or "working through" real-world challenges)." [3 p650]

"We offer these rules not as definitive guidance but as the starting point for further debate and empirical study. We invite others to test and improve them as they address value complexity in their own projects and settings." [3 p665]

We clarified this reflexive use more explicitly in the Methods section (Data analysis) and renamed the relevant discussion section to "A reflexive use of Greenhalgh et al.'s rules of thumb in CP development" to better reflect this interpretive stance. While we appreciate your suggestion to visualise the rules' application in a table or figure that systematically maps the rules onto our findings, we believe this would imply a level of prescriptiveness and completeness that contradicts the spirit of our interpretive approach.

DATA ANALYSIS: In phase 4, the research team consolidated the thematic structure. This process led to consensus on two overarching themes that reflected value complexity: goal alignment and decision-making in CP development. These were further analysed using value complexity[3] as an interpretive lens. Drawing on elements of Greenhalgh et al.'s[3] rules of thumb, they were not applied prescriptively but used to explore how tensions around values, goal alignment, and decision-making manifested in the data.
This approach guided a reflexive interpretation of the themes in relation to normative complexity.

Some where it is mentioned that the action research is a 2 year project, but it is not clear whether the funding also related to supporting the teams in their process and how the "ownership" of the project was embedded in the centre.

Thank you for raising this important point. The two-year action research programme was funded through a ZonMw grant, as part of the 'Actieonderzoek Innovatieve Zorg' funding scheme. The funding covered both the research activities and the facilitation of the CP development programme, including support for the participating teams. Ownership of the programme resided with the organisation's senior management and the embedded research team. This governance structure and the embedded role of the research team have now been clarified in the revised study settings and design section of the manuscript. We hope these revisions provide a more transparent account of how ownership and strategic alignment were secured throughout the programme.

STUDY SETTING AND DESIGN: see revised manuscript lines 134-162

The set of stake holders, and which of those were actually involved in the project is not clear.

Thank you for pointing out this omission in our paper. In the revised manuscript, we have clarified the stakeholders involved in or consulted during the CP development programme. While our data collection focused on members of the leadership triads (medical leaders, middle managers, and project leaders) and senior management - who were directly involved in the CP development work - we now also describe the broader stakeholder engagement that supported the programme. We have added this information to the Study settings and design section and referred to the visual overview in Figure 1. We hope this distinction between strategic involvement and direct participation clarifies the roles of the various stakeholder groups.

STUDY SETTING AND DESIGN: see revised manuscript lines 134-162

Further in the methods an extensive description is given on a consensus process, that is not traceable in a very structured way in the results.

We believe there may have been a misunderstanding regarding the use of the term consensus. In the Methods section, we refer to consensus within the research team during data analysis to indicate that interpretive agreement was reached through iterative discussion, as is common in qualitative reflective thematic analysis. This was not a stakeholder consensus process or formal consensus method such as Delphi. We have clarified this wording in the manuscript to avoid confusion.

DATA ANALYSIS: The thematic analysis was employed in accordance with Braun and Clarke's[29] six-phase methodology, blending inductive and deductive methods. In phase 1, the lead researcher familiarised herself with the participatory observation reports, identifying socially complex situations in CP development processes (e.g., conflict, diverging perspectives, or team demotivation) and the interventions deployed in response. These insights provided the foundation for generating the initial codes (phase 2). Next, the reflexive conversations and questionnaires were inductively coded by the lead researcher using Atlas.ti 24. All codes were reviewed and discussed in the research team to reach interpretive consensus.

In phase 3, the codes were grouped into four candidate themes. Conceptual maps were created in Atlas.ti to visualise thematic interconnections (see Appendices A and B). The

maps show how codes clustered into broader categories - such as financial viability, representation, and consensus building - and how these related to each other. These visualisations facilitated the research team's reflexive dialogue and iterative refinement of the thematic structure. This reflexivity extended beyond individual researcher's positioning to include reflection of how researcher perspectives shaped coding, theme development, and meaning attribution.[30]

It seems as if 8 steps (phases?, conversations) are reported upon, but it is not clear whether that leads to an actual consensus or conclusion, whether that was uniform or diverse etc. etc.

Thank you for raising this point. The "eight steps" you refer to correspond to the eight reflexive conversation rounds conducted during the AR programme. We have clarified this in the revised Data collection and participants section and now explicitly refer to these rounds in relation to Figure 1. The updated figure shows the timing of each round (Rounds I–VIII) in the data collection layer, highlighting how they were embedded in the programme structure. These rounds did not aim to reach formal consensus among participants but were intended to surface experiences, perspectives, and tensions during CP development. We hope this addition helps to clarify the iterative nature of the dialogue process and its contribution to the development of the findings. To avoid confusion, we now reserve the term cycle exclusively for the three CP development cycles within the AR programme and refer to the data collection rounds as reflexive conversation rounds throughout the manuscript.

DATA COLLECTION AND PARTICIPANTS: see revised manuscript lines 164-185

In between data and methods there are two passages on Trustworthiness and Value complexity reporting on issues that impress as experiences during the action research process. In relation to earlier remarks my advise would be to use this information to either describe the setting (when expected a priori) or to present them as results (when experienced ex post).
Organisational issue are always relevant in action research and it is not uncommon that financial issues play a role; it would be helpful to better distinguish the development, the research and managerial roles in the earlier project and action research descriptions.

Thank you for your question about our previous description may have created some ambiguity between experiential elements of the action research process and methodological rigour. We have clarified that the reflexive journal maintained by the lead researcher functioned both as a reflective tool during the research and as a formal source of participatory observational data and used for analysis. This clarification has been incorporated in the 'Research team and reflexivity' section. Although the structure of the 'Techniques to enhance trustworthiness' section remains unchanged, we carefully reviewed the phrasing to ensure that reflexivity is clearly framed as a strategy to enhance analytical rigour. Reflexivity was systematically integrated into the research process, in line with action research principles and qualitative quality criteria.

RESEARCH TEAM AND REFLEXIVITY: see revised manuscript lines 217-229

Later on in the paper there seems to be an emphasis on introducing "blended care" in the CPs. That is an issue worth investigating as such as it is known to be complex and brings its own

issues. Whether or not this was the case should be clear from the beginning (as part of the project description?) and rather deserves some specific background literature.

Thank you for this valuable remark. We agree that blended care is a relevant topic. In this paper, however, it serves as part of the broader innovation goal of the CP development programme, not as a primary focus of analysis. To clarify the role of blended care, we added a brief explanation in the study setting and design section. Also, we kept the explanation in the Results section to illustrate how initial innovation goals—including blended care—interacted with other priorities and contributed to value tensions.

STUDY SETTING AND DESIGN: see revised manuscript lines 134-162

ORIGINAL GOALS AND SET-UP: In the project's initiation phase, all stakeholders (senior management, board, and research team) agreed on a shared objective: employees would learn to develop CPs with smarter, more efficient processes to innovate care delivery and create uniformity across locations. At this stage, care quality, process improvement, innovation (including blended care), and financial viability were valued equally. A learning perspective was embedded through a quality collaborative setting, in which multidisciplinary teams were trained and guided in CP development. Each CP team was steered by a leadership triad, responsible for overseeing the development process and monitoring progress. The organisation was expected to gradually reduce its reliance on the researchers for guidance and project management, ultimately taking over full responsibility for the process.

The lack of structure or use of the rules to structure, the results, makes the conclusions and discussion difficult to follow. And advising the alike application of the rules of thumb without looking at alternatives is challenging. All in all a restructuring of the paper would improve the readability, logic's and generalizability. The discussion part can than be properly reviewed.

We agree that a prescriptive application of the rules of thumb would require a more structured presentation. However, our aim was not to structure the analysis or present the results according to each rule individually, but rather to use the rules as a reflexive framework to interpret emerging patterns related to value complexity. Our primary goal was to inductively study value tensions and then retrospectively interpret these through the lens of normative complexity. This is now made clearer in both the Methods (Data analysis) and Discussion (title and framing of the section on the rules of thumb). We hope this approach still offers a valuable empirical contribution to the application of the rules and supports future researchers in using them as a reflective tool.

DATA ANALYSIS: In phase 4, the research team consolidated the thematic structure. This process led to consensus on two overarching themes that reflected value complexity: goal alignment and decision-making in CP development. These were further analysed using value complexity[3] as an interpretive lens. Drawing on elements of Greenhalgh et al.'s[3] rules of thumb, they were not applied prescriptively but used to explore how tensions around values, goal alignment, and decision-making manifested in the data.
This approach guided a reflexive interpretation of the themes in relation to normative complexity.

DISCUSSION: A reflexive use of Greenhalgh et al.'s rules of thumb in CP development

It is not clear why the patients perspective is not taken into account, as there is a lot of literature on patient involvement in CP development. This should especially be relevant in rehabilitation.

Thank you for highlighting this point. We fully agree that the patient perspective is essential in care pathway development. In the action research programme in which this sub-study was embedded, patients were indeed invited to participate in CP development teams. However, our recently published paper on this topic[31]shows that only a minority of teams heeded this invitation. In our discourse analysis, we explored professionals' and managers' beliefs that explain why patient involvement was often postponed, limited, or avoided in practice.

This paper focusses on the value dynamics among professionals, exploring how tensions around goal alignment and decision-making emerge during CP development. We have clarified this scope in the Patient and Public Involvement and Limitations sections, and explicitly acknowledged the absence of patient perspectives as a limitation. We also recommend this as an area for future research.

PATIENT AND PUBLIC INVOLVEMENT: Patients and members of the public were not involved in the design, or conduct, or reporting, or dissemination plans of this study. This decision reflects the study's focus on professionals' perspective and their engagement in reflexive dialogues on goal alignment and decision-making. However, in the broader action research programme in which this sub-study was embedded, patients were actively involved as stakeholders in several CP development teams.[31]

Lastly style can be improved, sometimes the authors use very long sentences (e.g. 123-127) and there some typo´s.

Thank you for this stylistic suggestion. We carefully reviewed the manuscript to identify and revise long or complex sentences. We also performed a final spellcheck to correct any remaining typographical errors.

Reviewer 2: Dr. Jean-Baptiste Gartner

Thank you for your constructive and encouraging review. Your suggestions greatly helped us sharpen the conceptual framing of value complexity, improve transparency in our methods, and strengthen the connection between data and interpretation. We have addressed your comments point by point below.

Comments to the Author

The theme is extremely relevant and interesting, and the very comprehensive literature on the concept of care pathways demonstrates a detailed understanding of the field's knowledge. However, you could expand on your thoughts and your introduction to the concept of value in healthcare. Here are a few suggestions that might strengthen your introduction:

- Sturmberg, J. P. (Ed.). (2016). The value of systems and complexity sciences for healthcare (No. 8801). Switzerland: Springer International Publishing.
- Sturmberg, J. P. (2018). Health system redesign. How to make health care person-centered, equitable, and sustainable. Cham: Springer.

We agree that the introduction of the concept of 'value' in healthcare could benefit from a broader conceptual framing. In the revised introduction, we now elaborate on the meaning of value in healthcare from a complexity-informed perspective, drawing on recent literature that frames value as emergent, negotiated, and context-dependent.

INTRODUCTION: In parallel, a growing body of research promotes the application of complexity thinking to healthcare (quality) improvement. Complexity is defined as 'a dynamic and constantly emerging set of processes and objects that not only interact with each other, but come to be defined by those interactions'.[1 p.42] This perspective challenges the prevailing linear thinking and emphasises the need to embrace unpredictability in improving health services.[2-4] Scholars conceptualise healthcare organisations as complex adaptive systems, characterised by non-linear dynamics, distributed agency, and context-sensitive learning.[2,5]

Contributing to this complexity is the presence of multiple, often conflicting values – such as autonomy, care quality, and efficiency - that actors must navigate in quality improvement efforts.[6-11] Greenhalgh et al.[3] capture this complexity with the concept of value complexity (or normative complexity[6]), defined as 'the complexity arising from differences in people's worldviews, interests, and values, leading to mistrust, misunderstanding, and conflict among stakeholders'.[3 p648] In line with other scholars[6,8,10,11], this definition acknowledges that value is not fixed or predefined, but emergent and context-dependent - shaped through ongoing interactions, reflective deliberation among professionals, patients, and managers. To support moral reasoning and reflection on value complexity, Greenhalgh et al.[3] propose ten rules of thumb (see Table 1), derived from diverse research traditions. These rules aren't intended as prescriptive checklists, but as flexible, context-sensitive starting points for engaging with value complexity in empirical research. The rules encourage users to critically reflect on themes as power dynamics, the use of numbers and visual representations, and uncertainty within complex moral landscapes.

Formulation of the research question: as an interpretivist paradigm methodology, it seems counterproductive to 'limit' yourselves to the normative framework of Greenhalgh et al. In my opinion, the research question should have been more open, leaving space for other themes to emerge; and runs counter to systems thinking and the science of complexity (which leaves room for emergence, in particular)

Thank you for raising this point. We recognise the tension between applying a normative framework and the principles of interpretivist and complexity-informed research. In our study, we used the rules as a reflexive lens during the later stages of analysis, using them to deepen our interpretation of how value tensions around goal alignment, and decision-making manifested in the data. Rather than applying the rules prescriptively or as coding categories, we drew on them to guide normative reflection and sense-making. This interpretive and reflexive stance aligns with Greenhalgh et al.'s intent:

"We sought to present this theory not as fixed predictive statements but as a set of heuristics or rules of thumb—that is, points of departure for reflection and situated moral reasoning ("thinking through" or "working through" real-world challenges)." [3 p650]

"We offer these rules not as definitive guidance but as the starting point for further debate and empirical study. We invite others to test and improve them as they address value complexity in their own projects and settings." [3 p665]

We clarified this reflexive use more explicitly in the Methods section (under Data analysis) and renamed the relevant discussion section to "A reflexive use of Greenhalgh et al.'s rules of thumb in CP development" to better reflect this interpretive stance. Also, in the introduction we have rephrased the research aim and rationale to clarify that the Greenhalgh et al. 'rules of thumb' were used as a reflexive lens rather than a prescriptive framework. Our revised text now underscores the openness to emergent themes during data analysis. We hope this approach offers a nuanced and transparent contribution to the empirical exploration of normative complexity, as called for by Greenhalgh et al.

INTRODUCTION: [..] Against this background, this study explores how CP development unfolds in practice, with particular attention to tensions that emerge as stakeholders navigate competing goals, values, and interests. […]

Value complexity is relevant in CP development, where diverse perspectives must be negotiated and integrated - a challenge that our study seeks to address. In doing so, this study responds to Greenhalgh et al.'s[3] call for further reflection and empirical study of their rules of thumb, using them as a reflexive lens in a CP development programme. The research question guiding this study was: "How does value complexity unfold in a CP development programme?"

DATA ANALYSIS: In phase 4, the research team consolidated the thematic structure. This process led to consensus on two overarching themes that reflected value complexity: goal alignment and decision-making in CP development. These were further analysed using value complexity[3] as an interpretive lens. Drawing on elements of Greenhalgh et al.'s[3] rules of thumb, they were not applied prescriptively but used to explore how tensions around values, goal alignment, and decision-making manifested in the data.
This approach guided a reflexive interpretation of the themes in relation to normative complexity.

Methods

The methodology section is well-constructed, but certain points need to be strengthened:

a) Research team and reflexivity: The reflexivity of researchers is a key point of credibility in qualitative research. The methods used should be better described and based on the methodological literature on qualitative research. See for example: Denzin, N. K., & Lincoln, Y. S. (Eds.). (2011). The Sage handbook of qualitative research. Sage

We agree that reflexivity is crucial in qualitative research, particularly in action research. Bradbury et al. identify reflexivity as one of seven key principles for enhancing and safeguarding the quality of action research. In line with their call to "take a personal, involved, and self-critical stance throughout the action research process, addressing the context of their research, their own identities and biases and how they might be received by participants, and what led to their involvement in this research", we have

explicitly reflected in this paper on what we learned through interactions with participants, as well as on the relationships and experiences that shaped the research. In the revised manuscript, we have expanded the description of the reflexive practices of the research team. We added references on this to strengthen the methodological foundation.

RESEARCH TEAM AND REFLEXIVITY: Reflexivity was an integral part of the methodological approach. Throughout the programme, the research team applied three- level reflexivity[23,25] to critically examine their own assumptions and prevent 'going native'.[26-28] This included: (1) the lead researcher maintaining a weekly reflexive journal, which served as a structured log of participatory observations used for both analytical and reflexive purposes capturing field-level complexity and supporting critical reflection on role tensions, researcher impact, and emergent value framings; (2) regular reflexive meetings within the research team to critically interrogate their interpretations, power dynamics, and interactions with participants, and (3) two moments of academic reflection within an academic advisory board convened by the University. This board was chaired by a professor from an outsider stance, and included the research team and rotating guests from the studied organisation, and provided a space for critical dialogue and methodological reflection. These activities are represented in Figure 1.

b) Data collection and sampling: The material mobilised seems rich and dense. A table or figure representing the material would be relevant. In addition, in order to reflect the evolution of the project over 2 years and the dynamic aspect of the controversies, the table/figure should show the chronology of the information gathered.

Thank you for this helpful suggestion. In response, we have taken several steps to improve clarity and transparency. First, we revised the Study settings and design section to include a clearer description of the two-year action research (AR) programme, including its original structure, the role and composition of the embedded research team, and the broader engagement of organisational stakeholders. We also added Figure 1, which visualises the longitudinal structure of the action research programme. The figure presents the three CP development cycles, key moments of data collection, and the evolving structure of stakeholder engagement over time. This timeline also provides insight into the intended sequencing of activities. Second, we expanded the Data collection and participants section to specify that participants in the reflexive dialogues and questionnaires included representatives from the roles in the leadership triad, as well as senior managers. Although participation varied slightly, all key stakeholder groups were represented across the dataset. Lastly, we refocused the Research team and reflexivity section to describe how reflexivity was applied to enhance analytical rigour, while information about the team's embedded role in the AR programme is now integrated in Study settings and design. We hope these adjustments enhance the structural coherence and contextual transparency of the manuscript.

STUDY SETTING AND DESIGN: This study was a sub-study of a two-year action research (AR) programme on CP development, conducted in a Dutch specialised rehabilitation hospital (16 sites, ~800 employees, ~16,000 patients annually). The programme was initiated by the centre's Executive Board and funded through a national AR grant scheme (ZonMw project number 05160062010008). A steering committee of senior management and medical leaders provided strategic oversight and alignment.

The AR programme aimed to support multidisciplinary teams in developing CPs that improved care quality, efficiency, and innovation - including the integration of blended care (i.e. the combination of face-to-face care with web-based digital interventions).[16- 18] In three CP development cycles, a total of eleven multidisciplinary teams were guided by an embedded research team in a quality collaborative approach[20], involving knowledge transfer, workshops and guided teamwork. Each multidisciplinary team was chaired by a leadership triad: a medical leader (rehabilitation physiatrist), a middle manager, and a CP project leader (healthcare professional, e.g., physiotherapist, speech therapist, or social worker).

The research team consisted of three members, and participated in the AR programme both as researchers and facilitators. The lead researcher coordinated the programme, coached the CP teams, and organised the collaborative meetings. The research team facilitated expert-led knowledge exchange, interactive exercises, and team-based learning throughout the collaborative meetings.

This paper reports a qualitative single-case study within this broader AR programme, following an interpretivist paradigm.[15,21,22] The sub-study followed a 'research- follow-action' strategy[23], in which action and learning occurred during the CP programme phase, while systematic analysis took place retrospectively. This approach allowed the research team to stay close to practice during facilitation, while maintaining interpretive distance in the analytical phase. Figure 1 visualises the structure of the AR programme, including the three CP development cycles, the timing of data collection activities and AR stakeholder meetings (see Figure 1).

Figure 2: AR programme timeline. This timeline illustrates the longitudinal set-up of the AR programme and data collection rounds. Data analysis was conducted retrospectively, following a research-follow-action strategy.

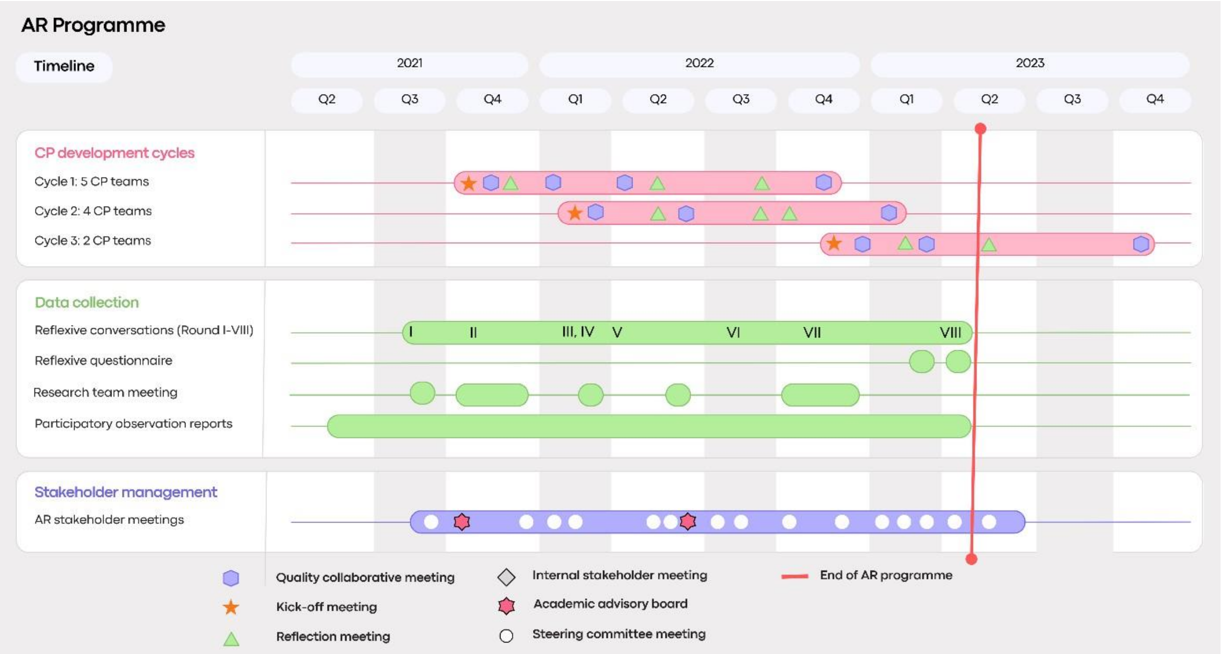

c) Data analysis: The analysis and coding process is very well explained (particularly with regard to techniques to enhance trustworthiness). However, you could add a word about coding to the paragraph on data analysis. In this paragraph, it is not clear whether the coding was carried out by at least two double-blind researchers or more. Nevertheless, the method seems very convincing.

We appreciate your remark. Although blind double-coding was not employed, all codes and emerging themes were critically reviewed and discussed in team sessions until consensus was reached. We mentioned this explicitly in the methods section to ensure that we inform the readers properly.

DATA ANALYSIS: The thematic analysis was employed in accordance with Braun and Clarke's[29] six-phase methodology, blending inductive and deductive methods. In phase 1, the lead researcher familiarised herself with the participatory observation reports, identifying socially complex situations in CP development processes (e.g., conflict, diverging perspectives, or team demotivation) and the interventions deployed in response. These insights provided the foundation for generating the initial codes (phase 2). Next, the reflexive conversations and questionnaires were inductively coded by the lead researcher using Atlas.ti 24. All codes were reviewed and discussed in the research team to reach interpretive consensus.

In phase 3, the codes were grouped into four candidate themes. Conceptual maps were created in Atlas.ti to visualise thematic interconnections (see Appendices A and B). The maps show how codes clustered into broader categories - such as financial viability, representation, and consensus building - and how these related to each other. These visualisations facilitated the research team's reflexive dialogue and iterative refinement of the thematic structure. This reflexivity extended beyond individual researcher's

positioning to include reflection of how researcher perspectives shaped coding, theme development, and meaning attribution.[30]

Further, please see revised manuscript lines 206-215.

d) Minor points: I don't think 'Patient and public involvement' is in the right place.

Thank you for your observation. As suggested, we reviewed the placement and content of our Patient and Public Involvement (PPI) statement. The PPI paragraph is located at the end of the Methods section, in line with BMJ Open guidelines. To further improve clarity, we revised the wording to explicitly state that no patient or public involvement occurred in the design or conduct of this sub-study, while also acknowledging the role of patient stakeholders in the broader action research programme. We believe the revised version better reflects the scope of the study and meets the journal's reporting standards.

PATIENT AND PUBLIC INVOLVEMENT: Patients and members of the public were not involved in the design, or conduct, or reporting, or dissemination plans of this study. This decision reflects the study's focus on professionals' perspective and their engagement in reflexive dialogues on goal alignment and decision-making. However, in the broader action research programme in which this sub-study was embedded, patients were actively involved as stakeholders in several CP development teams.[31]

RESULTS

Overall, the results are very interesting. But at this stage, the proposition still needs to be strengthened to convince the reader.

The shift in value linked to the quality of care towards the economic approach to value is a classic in healthcare improvement project. At this stage, the demonstration needs to rely even more on the hardware. For example, the verbatims are mainly (if not exclusively) from senior managers. Yet it is the perspective of middle managers and care professionals that should be presented to support the existence of the controversy (and remove the feeling that this controversy is primarily felt by the researchers themselves, reflected, for example, in the phrase: "This negatively affected the commitment of CP team members, who felt caught between two assignments: developing and implementing CPs").

Thank you for your question. We agree that presenting the voices of middle managers and care professionals is essential to substantiate the reported tensions and avoid the impression that these concerns were primarily researcher interpretations. In response, we reviewed our data excerpts and added quotes from CP project leaders, and a medical leader to more fully represent how these dynamics were experienced across different roles within the CP development teams. These additions show how production pressure and lack of time affected engagement at various levels, contributing to a loss of momentum and shared commitment in the project. This more clearly supports the controversy around competing priorities in care pathway development.

RESULTS: […] In the second shift in response to the financial crisis, management focused on enhancing short-term care productivity to drive revenue generation. This emphasis on increased productivity influenced how management directed employees which, in turn, reduced the number of CPs developed. Despite formal approval and funding through an external subsidy, management limited the hours employees could spend on the CP project. This negatively affected the commitment of CP team members, who felt caught between two assignments: developing and implementing CPs and maintaining or increasing care productivity. This dual responsibility left them no time for project work which delayed the development process. As one CP project leader put it:

"You feel the consequence of reducing the frequency of our meetings. We planned to alternate between meetings and working weeks, but that working time is gone. And now you feel like you're not on the moving train anymore—you keep stepping on and off the platform, trying to catch up" (CP project leader, I_40)

Team members at different levels echoed this tension. A coordinator described how shifting priorities eroded energy and a sense of shared commitment:

"Patient care took priority, and after a while there just wasn't much energy left. I really felt like I was standing alone." (CP project leader, I_41)

A medical leader reflected on how daily pressures overshadowed CP work and made participation difficult:

"It's incredibly busy, and there's a lot of pressure to fill the gaps, especially for patient- related tasks. There are so many things to juggle, and this [mid-May] was the first available moment since early April to work on this—it took a month and a half." (Medical leader, I_38)

The part about decision-making is fundamental for me. That is to say, can the moral values of the actors converge to make a constructive decision for the CP (and its actors), or can they not converge and so it is a question of power games and domination in decision-making.

We agree that the dynamics of moral values in decision-making are central. We have elaborated on this theme in the discussion, reflecting on whether decisions arose from mutual alignment or strategic dominance, and how this relates to normative complexity.

DISCUSSION: The key findings and their implications described above suggest that decision-making and goal alignment in CP development is not only a technical or procedural activity, but also a moral process shaped by divergent values and interests. In some teams, shared moral ground was actively cultivated through dialogue and iterative experimentation, allowing actors to align sufficiently to constructively make decisions. In other teams, however, alignment on values and interests became constrained due to strategic positioning or lack of managerial authority. This was particularly evident when financial pressure increased, which impeded decision-making. Value convergence requires active facilitation, it cannot be assumed to emerge naturally. This aligns with normative complexity, which refers to the coexistence of conflicting but legitimate values, such as patient-centredness, efficiency, and professional autonomy. These conflicting values complicate or even obstruct efforts at value convergence.[3,6,10] Rather than seeking full alignment, decision-making in such contexts requires recognition of moral tensions and facilitation of dialogical spaces

where diverse perspectives can be expressed, questioned, and negotiated. In this view, value conflicts are not problems to be resolved, but realities to be surfaced and engaged with.

From my point of view, the patient's view of what is valuable is the missing angle in the proposal.

We fully agree that the patient perspective is essential in care pathway development. In the action research programme in which this sub-study was embedded, patients were indeed invited to participate in CP development teams. However, our recently published paper on this topic[31] shows that only a minority of teams heeded this invitation. In our discourse analysis, we explored professionals' and managers' beliefs that explain why patient involvement was often postponed, limited, or avoided in practice. This paper focusses on the value dynamics among professionals, exploring how tensions around goal alignment and decision-making emerge during CP development. We have clarified this scope in the Patient and Public Involvement and Limitations sections, and explicitly acknowledged the absence of patient perspectives as a limitation. We also recommend this as an area for future research.

PATIENT AND PUBLIC INVOLVEMENT: see revised manuscript lines 248-252

LIMITATIONS: Finally, although patients were involved in the AR programme, they were not included in the data collection or analysis of this sub-study. As patient experience is a key dimension of value in healthcare, future studies should explore how patients perceive and contribute to goal alignment and decision-making.

DISCUSSION

The discussion is particularly relevant and some references (in particular Wehrens et al.) could have enriched your introduction (which remains too limited on value and complexity).

We elaborated more on value complexity in the introduction. By adding this information, we aim to provide more conceptual depth regarding the notion of 'value' from a complexity-informed perspective on value as introduced by Greenhalgh et al. Also, in the revised introduction, we now explain our rationale for choosing the Greenhalgh et al.'s rules of thumb. Rather than presenting it as prescriptive or even predictable framework, we introduce it as a reflexive tool aligned with our interpretivist and complexity-informed approach. Furthermore, we incorporated recent scholarship in both the introduction and the discussion.

The limitations are clear and well presented.

CONCLUSION

The conclusion is sufficient and interesting. I remain convinced that the Greenhalgh et al. framework should not be used as a normative framework but as a reflexive framework (which is what you say, by the way). This is therefore a second research perspective: to develop a framework of moral values in healthcare or to strengthen the one of Greenhalgh et al.

We fully agree with your interpretation. As clarified, the rules of thumb by Greenhalgh et al. were used to structure reflexive engagement rather than impose a normative blueprint. We have made this interpretive use more explicit in the revised Data analysis section and in the opening of the relevant Discussion section titled "A reflexive use of Greenhalgh et al.'s rules of thumb in

CP development". As you suggest, we also believe that further empirical research from this perspective, can develop the notion of complexity via moral values in healthcare.

DATA ANALYSIS: see revised manuscript lines 2063-212.

DISCUSSION: Greenhalgh et al.'s[3] rules of thumb (see Table 1) were used as a reflexive lens to interpret how value tensions emerged around goal alignment, and decision- making in CP development. This interpretive stance supported exploration of the moral and relational dimensions of these processes in a context-sensitive manner, consistent with the intended purpose of the rules.[…]

Future research is needed to explore how the rules of thumb can be used across diverse healthcare settings and to further develop practical strategies that support reflexivity, value exnovation, and 'working with' value complexity in complex change processes.

CONCLUSION: We encourage further research on value complexity both in and beyond CP development in (other types of) healthcare organisations. This includes exploring how reflexive approaches – such as Greenhalgh et al.'s rules of thumb – can be strengthened and translated into practical strategies to enhance reflexivity and dialogue among stakeholders to further support effective decision-making and goal alignment in CP development.

REFERENCES

BMJ open guidelines recommend to put DOI in the format : "doi: 10.xxxxxx" for all articles.

Thank you for this formatting reminder. We have revised the reference list to ensure that all journal articles now include the DOI in the format "doi: 10.xxxxxx", in line with BMJ Open guidelines.

IN BRIEF:

The overall objective has certainly been achieved. Overall, the manuscript is already of good quality and well written. Like any manuscript, it could be improved by reviewing the literature in the introduction section, improving the methodology section by producing a figure showing the data materials, reinforcing in the results section the role of the material in supporting the inferences made, in particular by cross-referencing the perspectives of the different categories of players involved. The results are overall relevant and interesting.

Good luck for the next steps.

Thank you very much for your positive and constructive assessment. We are grateful for your acknowledgement of the manuscript's overall quality and clarity, and appreciate your helpful suggestions for further improvement. As outlined in our earlier responses, we have reviewed and expanded the introduction, clarified and visualised the methodology using a new figure, and strengthened the results section by better linking data to interpretations and perspectives.

Reviewer 3: Dr. Wenjuan Tao

We thank you sincerely for your thoughtful and thorough review. Your suggestions have helped us to further clarify the methodological choices, enhance the transparency of our analytic process, and reflect on the transferability of our findings. We greatly value your perspective on the practical applicability of our findings and your attention to the presentation of materials. Please find our detailed responses to your points below.

Major Comments

1. The use of Greenhalgh et al.'s rules of thumb is a key strength, as it provides a structured lens to explore value complexity. However, the manuscript could better articulate how each rule was applied in the analysis. For instance, while the discussion references rules such as power dynamics (Rule 2) and language framing (Rule 4), the specific analytical process linking these rules to the data is somewhat unclear. Consider including a brief subsection in the Methods or Results section to clearly explain how each rule was operationalized during data analysis, such as indicating which codes or themes corresponded to specific rules. This would enhance transparency and replicability for readers unfamiliar with the rules.

Thank you for this valuable suggestion. We agree that greater clarity on the use of Greenhalgh et al.'s rules of thumb would enhance the transparency of our analytical approach. In our study, we used the rules not as a coding framework or prescriptive analytic model, but as a reflexive lens during the interpretive phase of analysis. This reflexive engagement allowed us to explore how normative tensions surfaced in relation to goal alignment and decision-making during CP development. To clarify this interpretive stance, we revised the final paragraph of the Data analysis section. We now explain more explicitly that the rules were used as sensitising concepts to guide reflexive dialogue in the research team, rather than being mapped directly onto specific codes or themes. This approach aligns with Greenhalgh et al.'s intent, who present the rules as heuristics for situated moral reasoning rather than a checklist for coding or evaluation:

"We sought to present this theory not as fixed predictive statements but as a set of heuristics or rules of thumb—that is, points of departure for reflection and situated moral reasoning." [3 p. 650]

"We offer these rules not as definitive guidance but as the starting point for further debate and empirical study. We invite others to test and improve them as they address value complexity in their own projects and settings." [3 p. 665]

We also renamed the relevant discussion subsection to "A reflexive use of Greenhalgh et al.'s rules of thumb in CP development" to better reflect this analytical positioning. While we considered your suggestion to create a table linking specific rules to codes or themes, we believe such a format would inadvertently imply prescriptiveness, which would be at odds with the interpretive nature of our approach. We hope the clarified explanation now supports a nuanced understanding of how the rules informed our engagement with normative complexity.

INTRODUCTION: Against this background, this study explores how CP development unfolds in practice, with particular attention to tensions that emerge as stakeholders navigate competing goals, values, and interests.[…]

Value complexity is relevant in CP development, where diverse perspectives must be negotiated and integrated - a challenge that our study seeks to address. In doing so, this study responds to Greenhalgh et al.'s [3] call for further reflection and empirical study of their rules of thumb, using them as a reflexive lens in a CP development programme. The research question guiding this study was: "How does value complexity unfold in a CP development programme?"

DATA ANALYSIS: In phase 4, the research team consolidated the thematic structure. This process led to consensus on two overarching themes that reflected value complexity: goal alignment and decision-making in CP development. These were further analysed using value complexity[3] as an interpretive lens. Drawing on elements of Greenhalgh et al.'s[3] rules of thumb, they were not applied prescriptively but used to explore how tensions around values, goal alignment, and decision-making manifested in the data.
This approach guided a reflexive interpretation of the themes in relation to normative complexity.

2. The single-case study design is noted as a limitation, which is appropriate, but the manuscript could do more to support transferability to other healthcare settings. While rich descriptions are provided, the discussion could explicitly address how the findings might apply to other contexts (e.g., acute care, primary care, or other rehabilitation settings).

Thank you for this constructive suggestion. While our study is context-specific, we agree it is important to reflect more explicitly on its transferability. In response, we expanded the 'Strengths and limitations' section to highlight how both the single-case nature and the specialised rehabilitation setting which may have shaped the observed value dynamics. We now clarify that the study took place in a Dutch specialised rehabilitation centre, where multidisciplinary collaboration and shared decision-making are embedded in daily practice.
These contextual features may have shaped the observed consensus-oriented dynamics. This is supported by earlier research showing how organisational culture influences team functioning in rehabilitation.[12,13]

STRENGTHS AND LIMITATIONS: As this is a single case study in one (multi-location) specialised rehabilitation hospital, transferability to other contexts may be constrained. Both the single-case nature and the specialised rehabilitation setting may have shaped the observed dynamics, as organisational culture is known to influence team functioning in such contexts.[12,13] To counter this effect, the researchers provide rich, detailed observations and interview excerpts to give other researchers the opportunity to assess the transferability of the findings.[15,21]

Minor Comments

1. "Value complexity" is central to this study, but the introduction only provides a definition of "complexity". I am still confused about what "value complexity" actually means.

Thank you for pointing out the need for more conceptual depth regarding the notion of 'value'. In the revised introduction, we now elaborate on the meaning of value in healthcare from a complexity-informed perspective, drawing on recent literature that frames value as emergent, negotiated, and shaped by context. This perspective aligns with our interpretivist approach and the study's focus on stakeholder interaction and moral reasoning. We also explain our rationale for choosing the Greenhalgh et al.'s rules of thumb. Rather than presenting it as the only available framework, we introduce it as a reflexive tool aligned with our interpretivist and complexity-informed approach.

INTRODUCTION: In parallel, a growing body of research promotes the application of complexity thinking to healthcare (quality) improvement. Complexity is defined as 'a dynamic and constantly emerging set of processes and objects that not only interact with each other, but come to be defined by those interactions'.[1 p.42] This perspective challenges the prevailing linear thinking and emphasises the need to embrace unpredictability in improving health services.[2-4] Scholars conceptualise healthcare organisations as complex adaptive systems, characterised by non-linear dynamics, distributed agency, and context-sensitive learning.[2,5]

Contributing to this complexity is the presence of multiple, often conflicting values – such as autonomy, care quality, and efficiency - that actors must navigate in quality improvement efforts.[6-11] Greenhalgh et al.[3] capture this complexity with the concept of value complexity (or normative complexity[6]), defined as 'the complexity arising from differences in people's worldviews, interests, and values, leading to mistrust, misunderstanding, and conflict among stakeholders'.[3 p648] In line with other scholars [6,8,10,11], this definition acknowledges that value is not fixed or predefined, but emergent and context-dependent - shaped through ongoing interactions, reflective deliberation among professionals, patients, and managers. To support moral reasoning and reflection on value complexity, Greenhalgh et al.[3] propose ten rules of thumb (see Table 1), derived from diverse research traditions. These rules aren't intended as prescriptive checklists, but as flexible, context-sensitive starting points for engaging with value complexity in empirical research. The rules encourage users to critically reflect on themes as power dynamics, the use of numbers and visual representations, and uncertainty within complex moral landscapes.

Value complexity is relevant in CP development, where diverse perspectives must be negotiated and integrated - a challenge that our study seeks to address. In doing so, this study responds to Greenhalgh et al.'s[3] call for further reflection and empirical study of their rules of thumb, using them as a reflexive lens in a CP development programme. The research question guiding this study was: "How does value complexity unfold in a CP development programme?"

2. The code networks in Appendices A and B are difficult to read and interpret. The manuscript would benefit from a brief description of what these code networks entail (e.g., key themes or relationships visualized).

Thank you for addressing this issue. We added brief descriptions of the code networks in the Results section to aid interpretation. These describe the thematic interconnections and how they informed the identification of the two main themes.

DATA ANALYSIS: In phase 3, the codes were grouped into four candidate themes. Conceptual maps were created in Atlas.ti to visualise thematic interconnections (see Appendices A and B). The maps show how codes clustered into broader categories - such as financial viability, representation, and consensus building - and how these related to each other. These visualisations facilitated the research team's reflexive dialogue and iterative refinement of the thematic structure. This reflexivity extended beyond individual researcher's positioning to include reflection of how researcher perspectives shaped coding, theme development, and meaning attribution.[30]

3. While the study offers valuable insights, the practical guidance remains somewhat abstract. The interventions described (reflexive dialogues, small-scale experimentation) need more detailed implementation guidance. What specific facilitation techniques were most effective? How might organizations without embedded action researchers implement these approaches?

Thank you for this constructive suggestion. We agree that practical implementation guidance on reflexive facilitation and small-scale experimentation is highly relevant to organisations seeking to apply similar approaches. However, assessing the effectivity of facilitation techniques was beyond the scope of this article. Our primary aim was to explore of value complexity in decision- making and goal alignment in CP development. We appreciate your point and intend to address these action-oriented learning and facilitation questions in future publications.

4. Table 2 & 3: Response rates vary considerably across cycles (50%-100%). Was there any pattern to non-participation that might introduce bias?

Thank you for raising this point. We have revisited the participation patterns and added further explanation to the Data collection section. While response rates varied (50–100%), no systematic non-response patterns were observed across the programme. Most important reason for non-participation was “due to time constraints”. Also, we added the possible bias in the strengths and limitations section.

DATA COLLECTION AND PARTICIPANTS: Participants for the reflexive conversations and questionnaires were selected through criterion sampling.[30] A total of 56 professionals and managers were invited by email, of whom 44 accepted (79% participation rate; see Table 2). The sample included representatives from all three roles in the leadership triad (N=21) as well as members of senior management (N=5). Each participant took part in one to four conversations, with an average length of 38 minutes. The first reflexive questionnaire, distributed to leadership triad representatives (N=25), received 14 responses (56% response rate), while the second, sent to senior management (N=5), yielded five responses (100% response rate) (see Table 3). Although participation and

response rates varied, no systematic patterns of selective non-response were observed and the most important reason for not participating was "due to time constraints".

STRENGTHS AND LIMITATIONS: Additionally, the strategy chosen for criterion sampling might have led to selection bias, especially as multiple interviews were conducted with the same respondents over time. This may have resulted in an overrepresentation of coordinators' perspectives, as they were often more consistently available for participation.

REFERENCES

[1] Cohn S, Clinch M, Bunn C, Stronge P. Entangled complexity: why complex interventions are just not complicated enough. J Health Serv Res Policy 2013 Jan 1;18(1):40–43. doi: 10.1258/jhsrp.2012.012036

[2] Plsek PE, Greenhalgh T. Complexity Science: The Challenge Of Complexity In Health Care. BMJ 2001 Sep 15;323(7313):625–628. doi: 10.1136/bmj.323.7313.625

[3] Greenhalgh T, Engebretsen E, Bal R, Kjellström S. Toward a Values-Informed Approach to Complexity in Health Care: Hermeneutic Review. Milbank Q 2023 Sep;101(3):646–674. doi: 10.1111/1468-0009.12656

[4] Greenhalgh T, Papoutsi C. Studying complexity in health services research: desperately seeking an overdue paradigm shift. BMC Med 2018 June 20;16(1):95. doi: 10.1186/s12916-018-1089-4

[5] Sturmberg JP. "Returning to Holism": An Imperative for the Twenty-First Century. The Value of Systems and Complexity Sciences for Healthcare Cham: Springer International Publishing; 2016. p. 3–19.

[6] Cribb A, Entwistle V, Mitchell P. Talking it better: conversations and normative complexity in healthcare improvement. Med Humanit 2022 Mar 1;48(1):85–93. doi: 10.1136/medhum-2020-012129

[7] Oldenhof L, Wehrens R, Bal R. Dealing With Conflicting Values in Policy Experiments: A New Pragmatist Approach. Adm Soc 2022 Oct;54(9):1736–1766. doi: 10.1177/00953997211069326

[8] Oldenhof L, Postma J, Putters K. On Justification Work: How Compromising Enables Public Managers to Deal with Conflicting Values. Public Adm Rev 2014 Jan;74(1):52–6310.1111/puar.12153.

[9] Oldenhof L, Petit-Steeghs V, Wehrens R, van Haperen S, Heerings M. Working With Value Complexity in Healthcare. Qual Health Res 2025 Apr 10:10497323251330999. doi: 10.1177/10497323251330999

[10] Wehrens R, Oldenhof L, Heerings M, Petit-Steeghs V, Haperen Sv, Bal R, et al. Integrating System Dynamics and Action Research: Towards a Consideration of Normative Complexity Comment on "Insights Gained From a Re-analysis of Five Improvement Cases in Healthcare Integrating System Dynamics Into Action Research". Int J Health Policy Manag 2023 Jan 1;12(Issue 1):7582. doi: 10.34172/ijhpm.2023.7582

[11] Wolf A, Erichsen Andersson A, Wikström E, Bååthe F. Untangling the perception of value in value-based healthcare – an interview study. LHS 2024 -04-16;37(5):130–141. doi: 10.1108/lhs-07-2023-0051

[12] Strasser DC, Smits SJ, Falconer JA, Herrin JS, Bowen SE. CLINICAL REPORT. Journal of Rehabilitation Research & Development 2002;39(1-4):115–125.

[13] Körner M, Wirtz MA, Bengel J, Göritz AS. Relationship of organizational culture, teamwork and job satisfaction in interprofessional teams. BMC Health Serv Res 2015 Jun 23;15(1):243. doi: 10.1186/s12913-015-0888-y

[14] Ellis BS. The value of systems and complexity thinking to enable change in adaptive healthcare organisations, supported by informatics. In: Sturmberg JP, editor. The value of systems and complexity sciences for healthcare Switzerland: Springer International Publishing; 2016. p. 217–229.

[15] Mortelmans D. Handboek kwalitatieve onderzoeksmethoden. 3rd ed. Leuven: Acco; 2020.

[16] Wentzel J, van der Vaart R, Bohlmeijer ET, van Gemert-Pijnen JEWC. Mixing Online and Face-to-Face Therapy: How to Benefit From Blended Care in Mental Health Care. JMIR Ment Health 2016 Feb 9;3(1):e9–e9. doi: 10.2196/mental.4534

[17] Toonders SAJ, Poolman EY, Nieboer ME, Pisters MF, Veenhof C. Healthcare professionals' perspectives on a blended care program in primary care; A qualitative study. Internet Interv 2021 Dec 1;26:100440. doi: 10.1016/j.invent.2021.100440

[18] Kip H, Wentzel J, Kelders SM. Shaping Blended Care: Adapting an Instrument to Support Therapists in Using eMental Health. JMIR Ment Health 2020 Nov 13;7(11):e24245. doi: 10.2196/24245

[19] Hohberg V, Fuchs R, Gerber M, Künzler D, Paganini S, Faude O. Blended Care Interventions to Promote Physical Activity: A Systematic Review of Randomized Controlled Trials. Sports Med - Open 2022 Dec 1;8(1):100. doi: 10.1186/s40798-022-00489-w

[20] Zamboni K, Baker U, Tyagi M, Schellenberg J, Hill Z, Hanson C. How and under what circumstances do quality improvement collaboratives lead to better outcomes? A systematic review. Implementation Sci 2020 May 4;15(1):27. doi: 10.1186/s13012-020-0978-z

[21] Green J, Thorogood N. Qualitative methods for health research. 4th edition ed. Los Angeles: SAGE; 2018.

[22] Roberts K, Casey M, Coghlan D, Cornall C, Hudson C, Stokes D, et al. A scoping review protocol to map the evidence on the use of action research methodology by healthcare professionals and in healthcare team settings. HRB Open Res 2021(4):68. doi: 10.12688/hrbopenres.13275.1

[23] Zandee DP, Coghlan D. Action research for impact in addressing the grand challenges. Strateg Organ 2025 Feb 1;23(1):147–161. doi: 10.1177/14761270241270913

[24] Schreier M. Sampling and Generalization. In: Flick U, editor. The SAGE Handbook of Qualitative Data Collection London; London: SAGE Publications Ltd; 2018. p. 84–97.

[25] Coghlan D. Action research as the social science of change and changing. Handbook of Research Methods in Organizational Change Cheltenham, UK: Edward Elgar Publishing; 2023. p. 19–46.

[26] Tresch J. On Going Native. Philos Soc Sci 2001 September 1;31(3):302–322. doi: 10.1177/004839310103100302

[27] Fuller D. Part of the action, or 'going native'? Learning to cope with the 'politics of integration'. Area (Oxf) 1999 Sep;31(3):221–227. doi: 10.1111/j.1475-4762.1999.tb00086.x

[28] Bradbury H, Glenzer K, Apgar M, Embury DC, Friedman V, Kjellström S, et al. Action Research Journal's seven quality choicepoints for action oriented research for transformations. Action Res (Lond) 2020 Mar 1;18(1):3–6. doi: 10.1177/1476750320904562

[29] Braun V, Clarke V. Using thematic analysis in psychology. Qual Res Psychol 2006;3(2):77–101. doi: 10.1191/1478088706qp063oa

[30] Braun V, Clarke V. Toward good practice in thematic analysis: Avoiding common problems and be(com)ing a knowing researcher. Int J Transgend Health 2023;24(1):1–6. doi: 10.1080/26895269.2022.2129597

[31] Visser M, 't Hart N, Mul M, Weggelaar - Jansen AM. The Perspectives of Healthcare Professionals and Managers on Patient Involvement in Care Pathway Development: A Discourse Analysis. Health Expect 2024 Jun;27(3):e14101–n/a. doi: 10.1111/hex.14101

VERSION 2 - REVIEW

Reviewer 1
Name van Harten, Wim
Affiliation University of Twente, Department of Health Technology and Services Research

Date **07-Jul-2025**

COI

The paper is much improved and now presents the method, material, setting, analysis and results in a more accessible way. On reading the revised text which now is much more consistent and understandable, also for an international audience, one issue remains that needs attention.

The CP development process is described as a complex process in which different values have to be balanced/negotiated. This seems to hypothesize that the underlying evidence base is either `given` (and further not mentioned) or negotiable as part of the values. One could however presume the evidence base of different strength per involved discipline, leading to different starting-positions in the discussion or (in extremis) shifting positions from strong i.e. evidence based to weak i.e. value based. Nor in the introduction, nor in the methods- or results section or in the discussion the role of evidence and its influence on values and the "relative power" of the involved discipline is being discussed. In the discussion the CP development literature is referred to, stating that "integrating best practices and evidence based care" should be pursued. This aspect is missed throughoyut the paper.

VERSION 2 - AUTHOR RESPONSE

RESPONSE TO THE REVIEWERS

Reviewer 1: Prof. dr. Wim van Harten

The paper is much improved and now presents the method, material, setting, analysis and results in a more accessible way. On reading the revised text which now is much more consistent and understandable, also for an international audience, one issue remains that needs attention.

Thank you for your thorough reading of our revised manuscript and for your positive feedback on its improved clarity and accessibility. Please find our response to your comment regarding the role of evidence in care pathway (CP) development, and the dynamics between evidence, values, and professional power below.

The CP development process is described as a complex process in which different values have to be balanced/negotiated. This seems to hypothesize that the underlying evidence base is either `given` (and further not mentioned) or negotiable as part of the values. One could however presume the evidence base of different strength per involved discipline, leading to different starting-positions in the discussion or (in extremis) shifting positions from strong i.e. evidence based to weak i.e. value based. Nor in the introduction, nor in the methods- or results section or in the discussion the role of evidence and its influence on values and the "relative power" of the involved discipline is being discussed. In the discussion the CP development

literature is referred to, stating that "integrating best practices and evidence based care" should be pursued. This aspect is missed throughout the paper.

Thank you for your comment on the role of evidence in CP development. In our study, CP teams were instructed to incorporate recent care innovations, professional treatment recommendations grounded in the available (evidence-based) guidelines into the design of their CPs. In several teams, these sources served as reference points for CP development. However, their application was not always straightforward. In the revised manuscript, we have expanded the fourth part of the results section on decision-making, where we describe how following professional guidelines sometimes implied a higher intensity of care than was feasible within the organisation's model for calculating care cost and reimbursement. In these cases, CP teams experienced tensions between clinically desirable content and financial viability. This constrained their decision-making and, in some cases, resulted in partial implementation of evidence informed guidelines. In the results section, we have revised the text as follows:

> *RESULTS: Some teams developed CPs using evidence-based guidelines or national treatment frameworks. However, applying these recommendations sometimes implied a higher frequency or longer duration of therapy than was financially feasible under the existing reimbursement model. This discrepancy became apparent when applying the organisation's calculation model, designed to assess the balance between care provision and expected reimbursement. As a result, tensions arose between clinically optimal care and financial viability. In such cases, CP teams had to weigh complex medical, ethical, and organisational considerations, which made the decision-making process more challenging*.

In addition, we have adjusted the in-text framing of CP development goals to clarify the literature's assumption that these goals are often seen as complementary and mutually reinforcing. While this point was originally included in the discussion, we have now moved it to the introduction. In the discussion, we now state that our findings challenge this assumption, showing that these goals may compete during CP development, particularly under organisational pressure.

> *INTRODUCTION: In addition, CP development pursues multiple goals simultaneously – enhancing patient experience, integrating evidence-based practices, and improving efficiency through standardisation and process optimisation – with the ultimate aim of improving team and patient outcomes.[1,4,10] These goals are assumed to be complementary and mutually reinforcing. [1,4,10,46-48] However, how these goals are prioritised and balanced in practice remains underexplored.*

> *DISCUSSION: While CP literature describes CP goals as complementary and assumes that goal alignment will naturally emerge over time to facilitate CP implementation, our findings show that this process is challenging.[7,49] While core values - patient-centred care, efficiency, and care-content improvement - remained stable, their prioritisation*

shifted throughout the CP development process, generating tensions and hindering alignment at both management and CP team levels. Rather than being complementary or reinforcing each other, goals frequently competed, particularly under organisational pressure, leading to misunderstanding, tensions in collaboration, and value conflicts.

Finally, we added a note to the Limitations section indicating that although CP teams were encouraged to incorporate evidence-based or evidence-informed guidelines when developing the CP, our study did not specifically examine how perceived variation in the strength of evidence across disciplines might have influenced goal alignment or decision-making.

LIMITATIONS: Finally, although CP teams were encouraged to incorporate evidence-based care, our study did not examine how perceived variation in evidence strength across disciplines influenced CP development. Future research could explore how such disciplinary differences shape value negotiation and influence decision-making processes.

We greatly appreciate your contribution to sharpening the analytical focus and positioning of our paper.

VERSION 3 - REVIEW

Reviewer 1

Name van Harten, Wim

Affiliation University of Twente, Department of Health Technology and Services Research

Date 28-Jul-2025

COI

The authors have addressed the remaining item in both results and discussion passages. I am somewhat surpised by the relative value that seems to be given to "evidence" in this case, but that might rather reflect the culture of the specific institution and its circumstances, than function as a generalizable statement. As this paper deals with this specific institution, this is acceptable.
